# Kinetics of large-scale chromosomal movement during asymmetric cell division in *Escherichia coli*

**DOI:** 10.1371/journal.pgen.1006638

**Published:** 2017-02-24

**Authors:** Jaana Männik, Matthew W. Bailey, Jordan C. O’Neill, Jaan Männik

**Affiliations:** 1 Department of Biochemistry and Cellular and Molecular Biology, University of Tennessee, Knoxville, Tennessee, United States of America; 2 Department of Physics and Astronomy, University of Tennessee, Knoxville, TN, United States of America; A*STAR, SINGAPORE

## Abstract

Coordination between cell division and chromosome replication is essential for a cell to produce viable progeny. In the commonly accepted view, *Escherichia coli* realize this coordination via the accurate positioning of its cell division apparatus relative to the nucleoids. However, *E*. *coli* lacking proper positioning of its cell division planes can still successfully propagate. Here, we characterize how these cells partition their chromosomes into daughters during such asymmetric divisions. Using quantitative time-lapse imaging, we show that DNA translocase, FtsK, can pump as much as 80% (3.7 Mb) of the chromosome between daughters at an average rate of 1700±800 bp/s. Pauses in DNA translocation are rare, and in no occasions did we observe reversals at experimental time scales of a few minutes. The majority of DNA movement occurs at the latest stages of cell division when the cell division protein ZipA has already dissociated from the septum, and the septum has closed to a narrow channel with a diameter much smaller than the resolution limit of the microscope (~250 nm). Our data suggest that the narrow constriction is necessary for effective translocation of DNA by FtsK.

## Author summary

DNA translocases are conserved throughout bacteria. While at atomic and molecular levels they have been well characterized, their ability to partition DNA in vegetatively growing cells has remained less clear. Here we show that *E*. *coli* translocase, FtsK, can move as much as 80% (3.7 Mb) of the chromosomal DNA across the closing septum in asymmetrically dividing cells at an average rate of 1700 bp/s. The majority of DNA movement occurs at the latest stages of cell division when the septum has closed to a narrow channel. Our data implies that a narrow septal opening is needed for effective translocation of DNA by FtsK.

## Introduction

Cell proliferation requires that each daughter cell inherits a complete set of chromosomes as a result of cell division. Consequently, proper spatial and temporal coordination between cell division proteins and chromosome replication and segregation is essential [1–3]. A high level of coordination is thought to be realized in many bacteria by precisely controlling the assembly of cell division proteins relative to chromosomes [4–6]. In *Escherichia coli* and most other bacteria, the assembly starts with the polymerization of FtsZ proteins into a filament network, the Z-ring, that attaches to the inner surface of the plasma membrane by ZipA and FtsA linker proteins [7–10]. The Z-ring and resulting septum are positioned very accurately at the center of rod shaped *E*. *coli* cells resulting in symmetric daughter cells [11–13].

Several partially redundant mechanisms have been discovered that are responsible for the localization of the Z-ring in *E*. *coli*. These mechanisms include nucleoid occlusion [2, 14], the Ter linkage [15], and the Min system [16]. Nucleoid occlusion in *E*. *coli* is mediated via SlmA, which inhibits Z-ring formation in the vicinity of the nucleoid [17]. Unlike nucleoid occlusion, which acts on Z-ring via negative regulation, the Ter linkage is a positive regulatory mechanism that promotes Z-ring formation near the replication terminus region of the chromosome (Ter). The Ter positions itself at the nucleoid center, which approximately coincides with the cell center, prior to completion of replication [18, 19]. ZapA, ZapB, and MatP have been implicated in realizing the Ter linkage [15]. While nucleoid occlusion and the Ter linkage rely on the spatial organization of the chromosome in the cell, the Min system positions the Z-ring independently of the chromosome [16]. Pole-to-pole oscillations of MinD and MinE, which are accompanied by the Z-ring inhibitory protein MinC, guarantee that cell division does not occur at cell poles. The Min proteins alone are also able to direct the Z-ring to cell center in nucleoid free minicells [20]. Taking that after replication the interchromosomal region positions itself at the cell center, the Min system usually guarantees a reliable positioning of the Z-ring relative to the chromosomes. Interestingly, all three Z-ring localization mechanisms, i.e. SlmA, positive regulation from the Ter region, and the Min system, can be removed from *E*. *coli* without loss of cell viability [15]. In the resulting cells, Z-rings frequently localize asymmetrically relative to the nucleoid and cell centers but the cells are able to grow and divide in slow growth conditions.

In addition to systems that coordinate Z-ring localization with the positioning of the chromosome, *E*. *coli* also possesses the DNA translocase, FtsK, which plays a role in chromosome segregation and dimer resolution [21, 22]. FtsK and its homologues are widely conserved throughout bacteria. These proteins consist of an N-terminal integral membrane domain, a long unstructured linker region (~650 aa in *E*. *coli*) and C-terminal motor domain. The latter assemble into a hexameric pore that pumps DNA in an ATP dependent manner. DNA pumping by FtsK is directional in *E*. *coli* due to KOPS (FtsK-orienting polar sequences) sequences [23], which in both chromosomal replichores direct FtsK movement towards the *dif* site, located in the replication terminus region. The pumping activity is independent of the N-terminal and unstructured linker domains of FtsK as shown by *in vitro* assays based on magnetic tweezers [24, 25] and DNA curtains [26–28]. The assays have shown very fast, instantaneous pumping rates by the FtsK C-terminal domain ranging from 5 kb/s at 20°C to 16 kb/s at 37°C [27]. As such, FtsK appears to be the fastest known DNA processing machine. *In vitro* assays have also shown that periods of fast pumping are interrupted by spontaneous reversals and dissociations from DNA. Interestingly, FtsK is able to displace and sometimes bypass many DNA binding proteins [26].

While much is known about FtsK interactions with DNA at the atomic and molecular scales, there is limited understanding of how FtsK functions in partitioning DNA in the cellular context when it is attached to the divisome. Recent studies have shown that FtsK is involved in the ordered segregation of the replication terminus region of the *E*. *coli* chromosome that spans about 400 kb around the *dif* site [29, 30]. The role of FtsK in chromosome segregation is likely more important in mutant cells, which are defective in Z-ring localization. In Min^-^ cells with a truncated FtsK C-terminal domain, Yu *et al*. found that the cells appeared to have DNA trapped by the closing septum [31]. Although the trapped DNA amount was not characterized, this finding implied that FtsK is needed to clear large portions of chromosomal DNA from the septal region in cells with mislocalized division planes. A much larger role of FtsK is also implied from studies of sporulating *B*. *subtilis* cells that harbor an FtsK ortholog SpoIIIE. During sporulation about 70% of the chromosome is typically pumped from the mother cell compartment to the forespore [32]. Although *E*. *coli* does not sporulate, the similarity between the translocase domains of *E*. *coli* and *B*. *subtilis* implies that FtsK may possess similar processivity as SpoIIIE.

Here we investigate how *E*. *coli* cells with asymmetrically placed cell division planes partition their chromosomes. Using quantitative fluorescence microscopy, we find that at very late stages of the cell cycle FtsK is capable of pumping as much as 80% of the chromosome (3.7 Mb of DNA) from one daughter compartment to another at an average rate of about 1700 bp/s. Within time resolution of our measurements (~5 min), the pumping appears to be continuous in the majority of the cells and is always unidirectional. Interestingly, the majority of DNA transport occurs only after the early arriving Z-ring components have dissociated from the division septum and cell constriction has closed to a narrow channel with diameter smaller than the resolution limit of an optical microscope (~250 nm). Our findings suggest that the narrow constriction is needed for effective translocation of DNA by FtsK.

## Results

### Chromosomal movement across the division plane in asymmetrically dividing cells

The Z-ring rarely assembles over unsegregated chromosomes in *E*. *coli*. In slow growth conditions, this is also true for strains that carry *slmA* and *min* single and double deletions [15]. Further removal of *zapA*, *zapB* or *matP* from the Δ*slmA* Δ*min* background is needed to observe Z-rings assemble over unsegregated nucleoids away from their centers [15]. To understand how chromosomes are partitioned when the cell division plane intersects an unsegregated nucleoid mass, we chose Δ*slmA* Δ*minC* Δ*zapB* strain for further studies. This strain shows a high frequency of asymmetric divisions and short cell lengths. Δ*slmA* Δ*minC* Δ*zapA* cells also show a high frequency of asymmetric divisions but cells in this strain are longer, while in Δ*slmA* Δ*minC* Δ*matP* asymmetric cell divisions occur less frequently. To follow the division process in Δ*slmA* Δ*minC* Δ*zapB* cells, we fused native HupA with mCherry and used time-lapse phase and fluorescent microscopy to image the strain. HupA binding to DNA is considered to be largely non-specific and has been extensively characterized before [19, 33, 34]. The cells were grown in M9 glycerol medium in slow growth conditions to guarantee the simplest possible DNA topology at the time of division.

**Fig 1:**
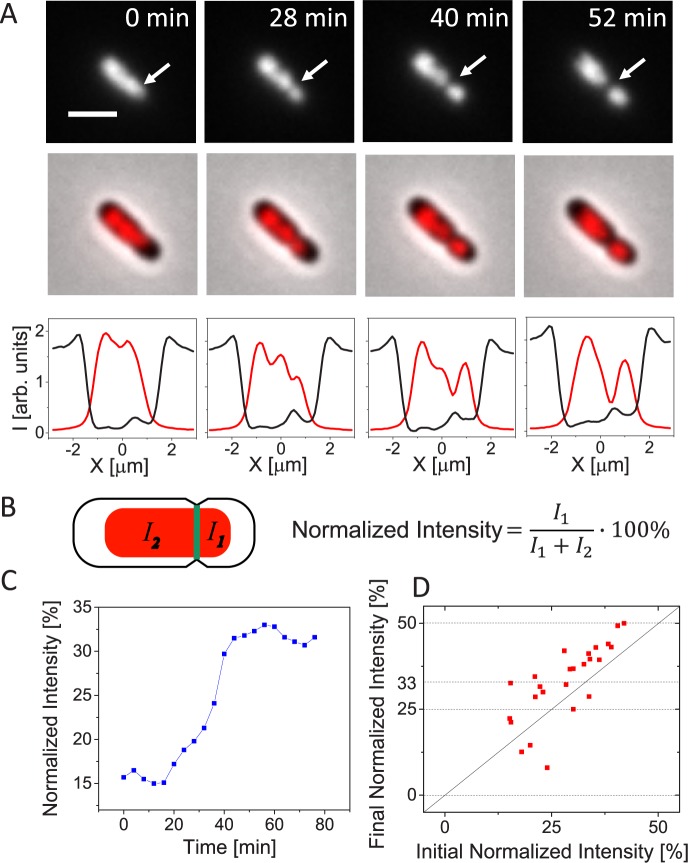
Chromosomal movement across the septum in asymmetrically dividing Δ*slmA* Δ*minC* Δ*zapB* cells. A: Time-lapse images showing HupA-mCherry labelled chromosomes (top row) and overlay of HupA-mCherry and phase images in an asymmetrically dividing cell (middle row). Bottom row shows intensity line profiles along the long axes of this cell. The black line is for phase and red for the HupA-mCherry profile. Time zero corresponds to the appearance of constrictions in phase images. Arrows in top row of images point to location of cell division plane. Strain JM30. Scale bar is 2 μm. B: Schematics showing calculation of normalized intensity. *I*_*1*_ and *I*_*2*_ are the integrated fluorescent intensities of the nucleoid label on the smaller and larger daughter side of the cell division plane, respectively. C: Normalized intensity from HupA-mCherry label during cell division for the cell shown in panel A. D: Initial normalized intensity at the time when constriction appears vs. final normalized intensity at the time when cells divide for 25 cells. Horizontal dotted lines show expected final normalized intensity values. Solid diagonal line marks no change in normalized intensity during division.

We first describe in detail chromosomal movement in a representative asymmetrically dividing Δ*slmA* Δ*minC* Δ*zapB* cell (Fig 1A) before discussing population wide data. In this and other asymmetrically dividing cells, there appeared to be no nucleoid occlusion effect, which would have prevented Z-rings from forming and constricting over unsegregated nucleoids. As a result of constriction, the underlying chromosome became pinched and trapped (Fig 1A, 0 and 28 min). As the constriction progressed, the trapped chromosome started to move from one daughter compartment to another (Fig 1A, 28–52 min, S1 Movie). After the movement completed, the two daughter cells separated. The daughters from this division show distinctly different lengths (1.2 μm and 2.4 μm, respectively). Despite different sizes, both daughters grew and underwent several rounds of divisions (S2 Movie). Viability of both daughters indicates that each must have inherited a complete chromosomal copy from their mother. To quantify the movement of the chromosome, we determined the normalized fluorescence intensity from the HupA-mCherry label on the smaller daughter side of the division plane (Fig 1B). At the time when the cell in Fig 1A started to constrict, its initial normalized intensity was 15% (Fig 1C). By the end of cell division, we would have expected the normalized intensity to rise to 50%, which would have corresponded to one complete chromosome in the smaller daughter compartment. Instead we found the normalized intensity to rise only to about 32%. Such a small increase appears inconsistent with the finding that the smaller daughter must have inherited a full chromosome and it also contradicts the apparent movement of chromosome in time-lapse images.

Smaller than expected final normalized intensity values were common for most cells studied (Fig 1D). All daughters from the divisions that we analyzed in Fig 1D grew and divided. We therefore expected the final normalized intensity values of 0, 25, 33 and 50% from these divisions. These expected values correspond to partitioning of zero or a positive integer number of complete chromosomes to each daughter cell when the mother cell had four or less chromosomes at the time of division. Specifically, the final normalized intensity value of 0% corresponds to divisions producing anucleate minicells, 25% to divisions that partition one chromosome to one and three to another daughter cell, and 33% to divisions that partition one chromosome to one and two to another daughter. Note that a mother cell yielding a normalized intensity of 33% arises when a cell with four chromosomes divides asymmetrically partitioning three chromosomes into one of the daughters. Comparing the measurement to the expected values of 0, 25, 33 and 50% (horizontal dashed lines in Fig 1D) showed that only in a few asymmetrically dividing cells did the data come close to these expected predictions. This finding implies that the change in the normalized intensity of HupA-mCherry signal does not reflect the true extent of the chromosomal movement in the cells.

### Quantifying chromosomal movement using different fluorescent reporters

**Fig 2:**
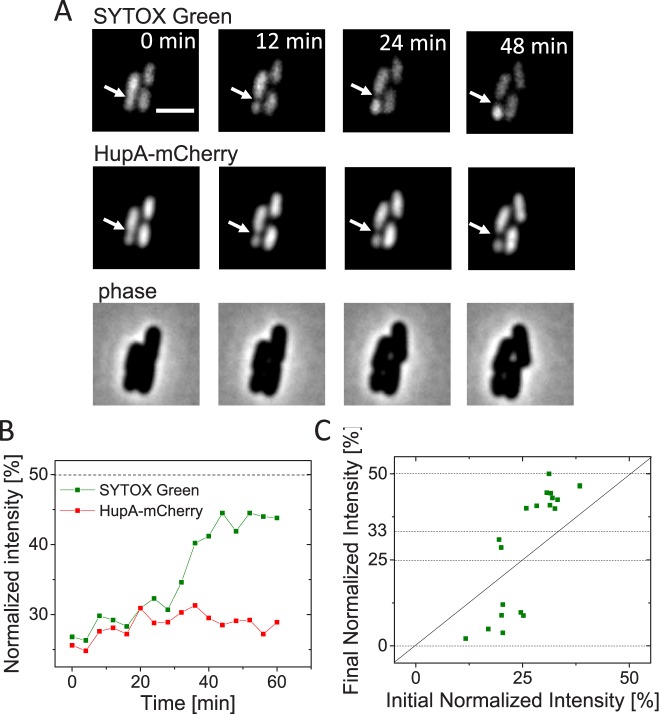
Chromosomal movement reported by SYTOX-Green and HupA-mCherry labels. A: Time-lapse images showing SYTOX-Green (top row) and HupA-mCherry labelled chromosomes (middle row) in a representative cell (strain JM30). Arrows point to location of cell division plane. Phase images for the same cell are shown in the bottom row. Scale bar is 2 μm. B: Normalized intensity from SYTOX-Green and HupA-mCherry labels during cell division for the cell shown in panel A. Dashed horizontal line corresponds to the expected value for both intensities at the end of the division. C: Initial normalized intensity vs final normalized intensity from SYTOX-Green label. Dashed lines correspond to the expected values for the final normalized intensity and solid diagonal to no change in intensity. N = 19.

We hypothesized that DNA movement in these cells is caused by the only known DNA translocase in *E*. *coli*, FtsK [21, 35, 36]. *In vitro* studies have shown that FtsK is capable of removing all tested DNA binding proteins except XerCD during DNA pumping [26]. Similar conclusions have also been reached in *in vivo* studies of SpoIIIE in sporulating *B*. *subtilis* [37]. Although previous studies did not specifically determine if FtsK is capable of removing HupA, this appears to be the likely outcome because of the relatively weak binding of HupA to DNA. To be able to quantify DNA movement during cell division we then tested if some membrane penetrable DNA dyes could be used for more reliable detection of DNA movement. In studies of DNA translocation during *B*. *subtilis* sporulation SYTOX-Green dye has been used [38, 39]. Indeed, after staining Δ*slmA* Δ*minC* Δ*zapB* cells with this dye we observed much more pronounced changes in the normalized intensity compared to what we found based on HupA-mCherry label (Fig 2A and 2B). The difference in changes of normalized intensity between these two labels is consistent with the idea that the HupA-mCherry label is removed during FtsK mediated pumping, and that after unbinding, HupA is not able to rebind to the DNA that was translocated. However, our data show that SYTOX-Green label is also partially removed during translocation albeit at much smaller extent than the HupA-mCherry label. We draw this conclusion from inspection of the final normalized intensities that still deviated significantly from the expected values of 50, 33, 25, and 0% (horizontal lines in Fig 2C). It is worth noting that deviations from expected final value (50%) were also apparent in earlier experiments in sporulating *B*. *subtilis* where SYTOX-Green reporter was used [38, 39].

**Fig 3:**
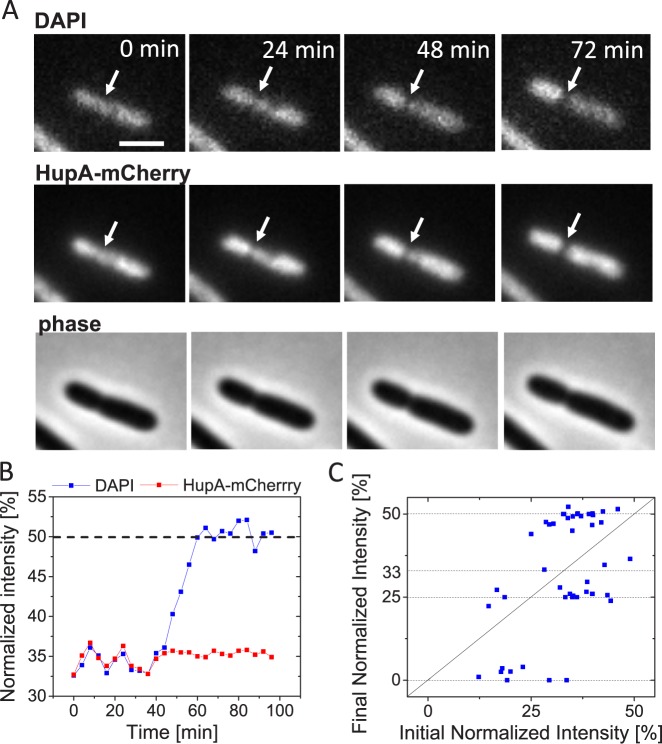
Chromosomal movement reported by DAPI and HupA-mCherry labels. A: Time-lapse images showing DAPI (top row) and HupA-mCherry labelled chromosomes (middle row). Arrows point to location of cell division plane. Phase images for the same cell are shown in the bottom row. Scale bar is 2 μm. B: Normalized intensity from DAPI and HupA-mCherry labels during cell division for the cell shown in panel A. Dashed horizontal line corresponds to the expected value for both intensities at the end of the division. C: Initial normalized intensity vs final normalized intensity from DAPI label (N = 46). Dashed lines correspond to the expected values for the final normalized intensity. In this plot data from cells without Z-ring marker (strain JM30) and with induced ZipA-GFP Z-ring marker (strain MB16) have been combined. The data from two strains are plotted separately in S3 Fig in Supplemental Materials. These data show no significant differences between the two strains.

Since SYTOX-Green still did not allow accurate quantification of DNA movement, we tested the suitability of DAPI labeling for these experiments. DAPI is only 277 Da in size, and it fits into minor groove of DNA without protruding out [40]. It seemed likely that DAPI could pass through FtsK hexameric pore without being removed. Although DAPI is not commonly used in time-lapse measurements our test indicated that it can be used for live cell fluorescence microscopy if binding density and light exposure are both low. Using exposures that were at least 1/8 weaker than for HupA-mCherry the majority of cells grew and divided. The growth rate at 28°C was about 25% lower in DAPI labelled cells (μ = 0.0028±0.0011 min^-1^; T_d_ = 248 min) compared to cells with only HupA-mCherry label (μ = 0.0038±0.0011 min^-1^; T_d_ = 182 min). Time-lapse imaging of DAPI and HupA-mCherry co-labelled cells (Fig 3A and 3B; S3 Movie) indeed confirmed that DAPI label allows quantification of DNA movement during translocation. We found that the final normalized intensity of the DAPI label reached very close to the expected values of 50, 33, 25 and 0% in almost all cells measured (Fig 3C) indicating that binding of the label to DNA is not significantly affected by the translocation.

In most cases, translocation curves based on DAPI labelled nucleoids reflected uninterrupted continuous change in intensity (as in Fig 3B) but in some cases pauses of 8 minutes (two frames) or longer could be detected (S1 Fig). Occurrence of such pauses were relatively rare (10% of all analyzed events). In no cases did we observe reversals of DNA movement (S3 Dataset and S4 Dataset in Supplemental Material). These findings indicate that at the time-scale of our measurements (~ 5 min) DNA movement is continuous and unidirectional. Similar conclusion could be also drawn based on measurements using HupA-mCherry and SYTOX-Green labels even though these measurements allowed limited quantification of DNA movement. Comparing HupA-mCherry tranlsocation traces to those of DAPI showed that in initial phases of translocation both normalized intensities evolved essentially identically but deviated from each other at final stages (S3 Dataset and S4 Dataset in Supplemental Material). HupA-mCherry and SYTOX-Green thus are able to indicate DNA movement at early stages of cell divisions but do not report it correctly in later stages.

### DNA movement is caused by FtsK translocation

**Fig 4:**
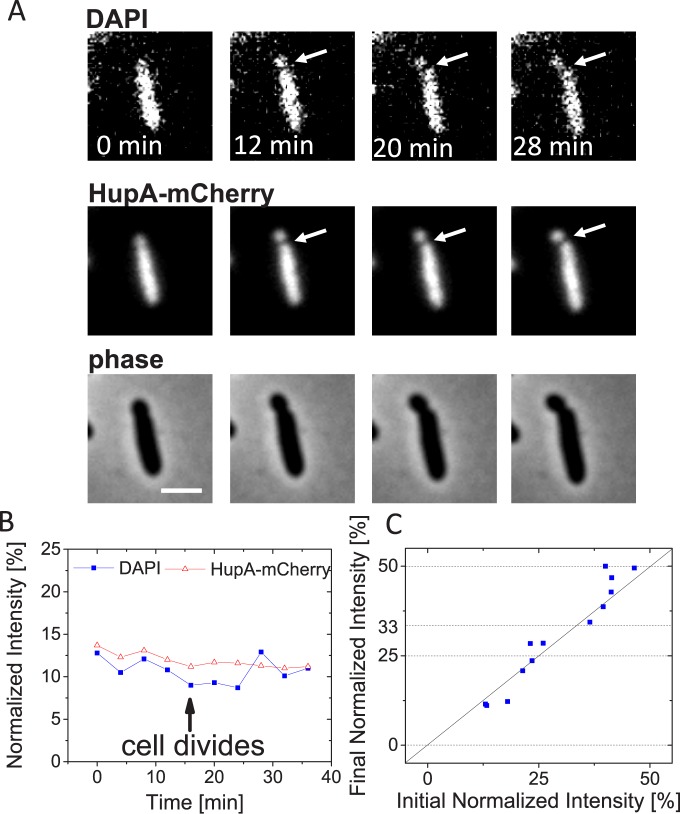
No large-scale chromosomal translocation in cells carrying additional FtsK K997A mutation. A: Time-lapse images showing DAPI (top row) and HupA-mCherry labelled chromosomes (middle row). Arrows point to location of cell division plane. Phase images for the same cell are shown in the bottom row. Scale bar is 2 μm. B: Normalized intensity from DAPI and HupA-mCherry labels during cell division for the cell shown in panel A. C: Initial vs final normalized intensity from DAPI label in strain JM38 (Δ*slmA* Δ*minC* Δ*zapB ftsK K997A*). Only cells whose length is smaller than 10 μm have been analyzed. Solid diagonal line corresponds to cases where the DNA amount in smaller daughter compartment remains unchanged during division. Dashed horizontal lines correspond to final normalized intensities of 0, 25, 33 and 50%. N = 13.

The previous experiments were suggestive that DNA movement during asymmetric divisions was caused by FtsK-mediated translocation, which removes HupA-mCherry, but leaves the DAPI label intact. To confirm the role of FtsK in DNA movement, we introduced an FtsK K997A mutation into Δ*slmA* Δ*minC* Δ*zapB* cells. The K997A mutation disables ATP binding to FtsK and abolishes DNA pumping [41, 42]. Remarkably, despite disabling all known coordination systems between cell division proteins and chromosomes [6], Δ*slmA* Δ*minC* Δ*zapB ftsK* K997A cells were still able to propagate in slow growth conditions (S4 Movie). However, some Δ*slmA* Δ*minC* Δ*zapB ftsK* K997A cells formed filamentous chains as have been reported for FtsK C-terminal mutants before [31, 43]. Also, time-lapse movies showed frequent lysing of cells after division (e.g. S4 Movie). This is in contrast to Δ*slmA* Δ*minC* Δ*zapB* strain where essentially all cells continued to grow and divide after division. It will require further studies to understand why Δ*slmA* Δ*minC* Δ*zapB ftsK* K997A cells lysed and to determine if some cells in this strain possibly cleaved chromosomal DNA. DNA cleaving/guillotining in cells defective in chromosome dimer resolution, as are *ftsK* K997A mutants, has been proposed earlier [44]. Importantly, neither HupA-mCherry nor the DAPI label in these cells showed any changes at the level observed for the FtsK active strain (Fig 4, S5 Movie, S5 Dataset in Supplemental Material, S5 Fig). The data thus further confirms that FtsK is the main factor responsible for the large-scale DNA movement in asymmetrically dividing Δ*slmA* Δ*min* Δ*zapB* cells.

### Estimation of DNA amount moving across the constriction and translocation speed

**Fig 5:**
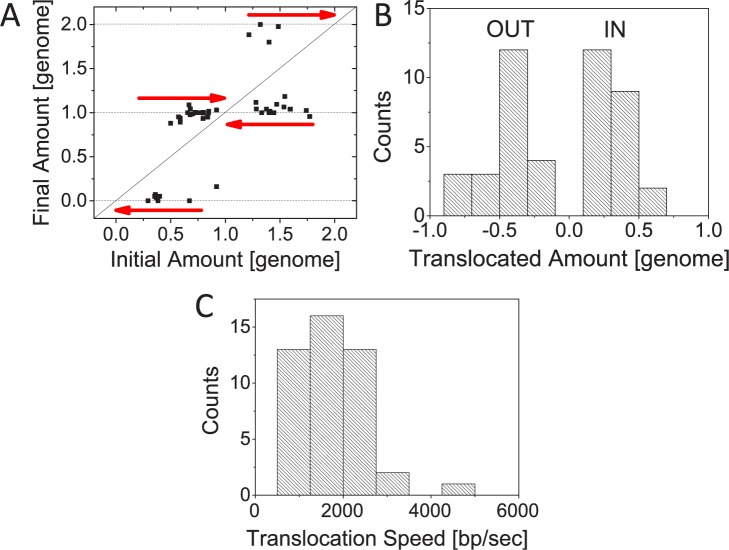
Quantification of DNA movement during translocation. A: Estimated amount of DNA in the smaller daughter compartment in the beginning and end of translocation. The amount of DNA is given in genome units (4.6 Mb). Arrows pointing to the right indicate divisions in which the DNA amount in the smaller daughter cell increases and arrows pointing to the left are those where the amount decreases. No change in DNA amount is marked by solid diagonal line. B: Distribution of the DNA amount that has crossed the division plane during translocation. DNA amounts are given in genome units. Positive amounts correspond to DNA moving into the smaller daughter compartment and negative amounts moving out from it. C: Distribution of translocation speeds. N = 46 for all the panels. Data from strains JM30 and MB16 is combined.

The data above have provided evidence that FtsK can translocate more than half of the *E*. *coli* complete chromosome (cf. Figs 1A, 3A and 3B). In what follows, we estimate the translocated DNA amount and the average translocation speed, and discuss directionality of DNA movement. In M9 glycerol medium, which is used to grow cells in these experiments, replication is expected to initiate only after the cell is born. Fully replicated DNA molecules should thus be present when cells divide. Consequently, we expect shorter cells to have two and longer cells (>4.5 μm; see Materials and Methods) four complete chromosomes at the time of translocation. The initial amount of DNA in the smaller daughter compartment at the beginning of translocation appears almost random reflecting the irregular positioning of the Z-ring in these cells. This initial amount can either increase or decrease (Fig 5A). If the smaller daughter compartment has less than a half of the chromosome in the beginning of the translocation then this chromosome portion is more likely removed from the daughter cell (Fig 5B). If the daughter has more than half of the chromosome then its missing chromosome portion is likely translocated to the cell. However, as Fig 5B shows, as much as 80% of DNA can leave or enter the smaller daughter compartment in some cases. From the duration of translocation we further estimate that the pumping occurs at an average translocation speed of 1700±800 bp/s (Fig 5C). In some cells translocation rates as high as 4200 bp/s can be observed. We did not find substantial correlation between the growth speed of cells and the translocation rate (S2 Fig; R = 0.06) indicating that any possible deleterious effects caused by DAPI label likely did not have a significant effect on translocation speed.

We also considered a possibility that in Δ*slmA* Δ*minC* Δ*zapB* mutant cells a well-defined cell cycle progression is lost and that partially replicated DNA molecules could be present at the time of division (S3 Fig). We used a cell length based estimate for DNA amount at the time of translocation. Using DAPI stained permeabilized cells, we found the scaling *DNA Amount* = 0.92(*L*_*cell*_ − 0.53) (for details see Materials and Methods). Here *DNA Amount* is in units of fully replicated chromosomes (of 4.6 Mb size) and cell length, *L*_*cell*_, is in micrometers. The formula indicates that at each pole there is 0.53/2 μm space without DNA and each nucleoid in the cell occupies about 0.92 μm of cell length. Using this estimate for the chromosomal amount at the time of translocation, we found that maximal amounts translocated reached upto 75% of genome (about 3.5 Mb) and the average translocation speed of 2100±900 bp/s (S3 Fig). These values are close to those calculated on assumption of fully replicated chromosomes being present at the time of translocation. Although FtsK in *E*. *coli* has been previously shown to affect only 400 kb region near replication terminus region in wild type cells [29, 30] our data shows that in conditions where the division plane is misplaced it is capable of moving as much as 3.5 Mb of DNA across closing division septum.

### Timing of DNA translocation

**Fig 6:**
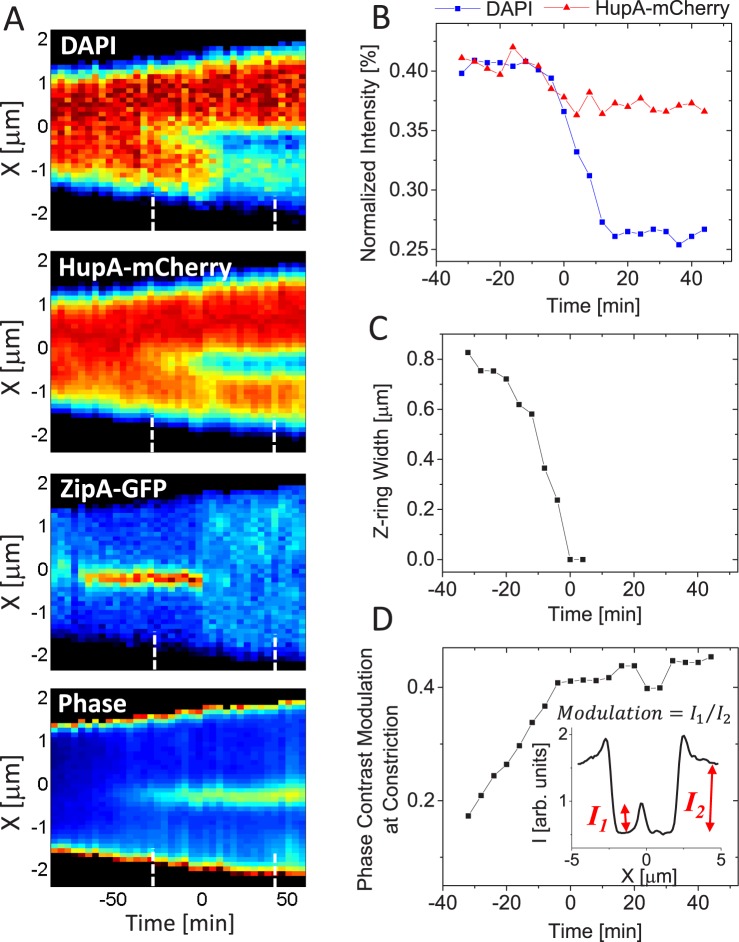
Determination of translocation timing based on Z-ring and phase contrast images. A: Kymographs of DAPI, HupA-mCherry, ZipA-GFP labels and phase contrast images along the long axes of a representative cell. Red corresponds to high and blue to low intensity. Black areas are outside the cell. Time zero corresponds to the first frame when no ZipA-GFP accumulation is present at the constriction. Dashed lines in the bottom of every kymograph show the time interval corresponding to traces in panels B-D. Strain MB16. B: Normalized intensity from DAPI and HupA-mCherry labels as a function of time for the same cell. C: Width of the Z-ring as a function of time. The width is measured along the short axes of the cell. D: Modulation of phase contrast images at the constriction as a function of time. The inset shows determination of the modulation from phase contrast profiles along the long axes of the cell.

The exact timing when FtsK mediated DNA translocation begins or ends has not been established in wild type or mutant *E*. *coli*. Our DNA translocation data allow determination of these times relative to septal closure. To monitor the septal closure we used ZipA-GFP as a Z-ring label. ZipA is an integral membrane protein that acts as one of the two membrane linkers for FtsZ. We also followed septal closure using phase contrast images. As in earlier measurements, DNA in the cells was labelled with both HupA-mCherry and DAPI. We first describe a division event in a representative cell before presenting data on a cell population. As can be seen from the kymographs in Fig 6A, the Z-ring forms slightly asymmetrically relative to cell center in this particular cell (distance 0.46L_cell_ from one of the poles). As described before, the ensuing constriction leads to the pinching of the chromosomal mass at the cell division plane but not to the immediate redistribution of DNA between the two daughter compartments. Only when the diameter of the Z-ring has decreased from about 0.8 μm to 0.4 μm is the translocation initiated (Fig 6B and 6C). Surprisingly, the majority of the DNA is translocated after the ZipA-GFP label has dissociated from the Z-ring. The translocation completes about 16 min after the label has dissociated from the division septum. During this last period, an estimated 2.2 Mb of DNA (of 3.0 Mb total) has crossed from the larger to the smaller daughter compartment. The dissociation of the ZipA-GFP label also coincides with the closure of the constriction as inferred from analysis of phase contrast images (Fig 6D). Although some connection between the two daughter compartments must be maintained for translocation, this connection appears so small that its disappearance before cells finally separate cannot be detected from phase contrast images. The width of the Z-ring before dissociation of ZipA-GFP (Fig 6C) indicates that the remaining connection has diameter much smaller than the extent of the point spread function of the microscope (~250 nm). The septal closure coincides with the time when the normalized intensity of the HupA-mCherry label settles to its final value. The latter indicates that the constriction remaining after ZipA label dissociates is small enough to effectively stop any diffusive flux of HupA-mCherry from one daughter compartment to another.

**Fig 7:**
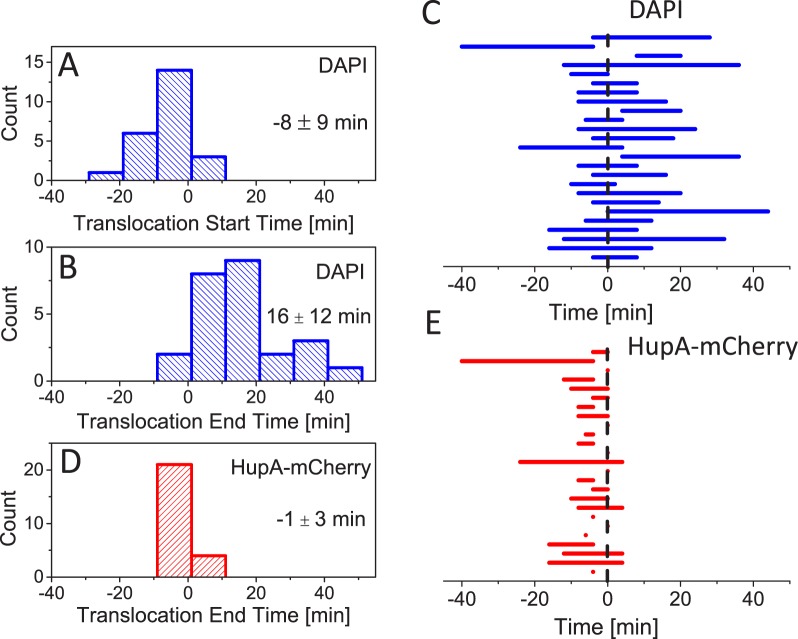
Translocation timing. A: Distribution of times when translocation starts as inferred from DAPI label. The times in all panels are referenced relative to the time when the ZipA-GFP label dissociates from the septum. Mean and std of distribution are indicated. Strain MB16. N = 24 for all panels. B: Distribution of times when translocation ends as inferred from DAPI label. C: Duration of translocation for individual cells as inferred from DAPI label. D: Distribution of times when HupA-mCherry normalized intensity stops to change. Note that HupA-mCherry normalized intensity starts to change at the same time as DAPI normalized intensity. E: Timing of translocation for individual cells as inferred from HupA-mCherry label.

Next we use the analysis described above for a population of cells to determine distributions of start and end times of translocation. From here on, time zero marks dissociation of ZipA-GFP from the septum (Fig 7). We find that on average translocation starts 8 minutes before the dissociation of ZipA-GFP from the septum (Fig 7A) and completes 16 minutes after the disappearance of the label (Fig 7B). We find large heterogeneity in timing but common to about 90% of cases, dissociation of the ZipA-GFP label occurs when translocation is ongoing (Fig 7C). In only 1 instance out of 26 (4%) did we observe translocation to complete before the label disappeared. In 3 out of 26 cases (12%) translocation started only after the label had disappeared. In cells whose translocation starts early relative to septal closure, HupA-mCherry is redistributed between daughter compartments and normalized intensity from this label follows that from DAPI. Redistribution of HupA-mCherry stops at about the time when ZipA-GFP label dissociates from the septum (Fig 7D and 7E). The abrupt stop in the change in the normalized intensity of HupA-mCherry is consistent with the idea that the constriction is sufficiently small to halt HupA-mCherry diffusion at the time when ZipA dissociates from the septum.

**Fig 8:**
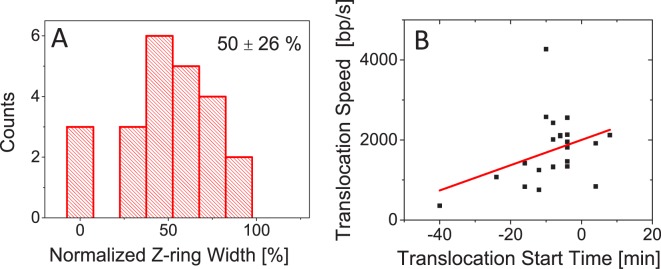
Correlations between translocation timing, width of the constriction, and translocation speed. A: Normalized width of the Z-ring when translocation initiates. For normalization, the width of unconstricted Z-ring is used. Mean and std of the distribution are shown. Strain MB16. N = 24. B: Translocation start time vs. translocation speed. Solid line is a linear fit (y = 32 x+2000; R = 0.37).

Lastly we also estimated to what extent the constriction had closed by the time translocation initiated. We found a wide distribution of constriction widths from essentially fully closed to constrictions that have decreased only by 10% from the unconstricted cell width (Fig 8A). On average, translocation initiates when the diameter of the constriction has decreased by about half (50±26%) from the unconstricted cell diameter. The time when translocation initiates affects translocation speed (Fig 8B). If the translocation initiates late then it progresses faster but the correlation between initiation time and translocation speed is relatively weak (R = 0.37). Altogether our data indicates that translocation occurs in very late stages of cell division when constriction has partially or even fully closed and that translocation speeds tend to increase the later the process initiates.

## Discussion

Removal of *slmA*, *minC* together with *zapA*, *zapB* or *matP* from the *E*. *coli* genome results in cells where Z-rings and division planes are frequently mispositioned relative to nucleoids [15]. In these cells, once the Z-ring forms and matures there appears to be no mechanism present to prevent the septum from constricting over underlying nucleoids and trapping them. We find that the DNA pump FtsK clears the trapped DNA from the constriction and partitions chromosomes to daughter cells in the final stages of cell division. Recent studies have provided evidence that FtsK translocates DNA in wild type *E*. *coli* only within the 200 kb region flanking the *dif*-site in the replication terminus region [29, 30]. Our data show that as much as 80% of the chromosome (3.7 Mb) can be translocated in Δ*slmA* Δ*minC* Δ*zapB* cells. It is possible that much smaller DNA movement was found in these previous works because FtsK acts in wild type cells on a smaller chromosomal region. The accurate positioning of the Z-ring in wildtype cells provides that there is no need for FtsK mediated partitioning of chromosomes unless chromosome dimers have formed. The largest chromosomal translocation amounts that we report here are similar to those found in sporulating *B*. *subtilis* cells (70% of genome) [32]. Also, the translocation speeds, 1700±800 bp/s from our measurements and 1000±160 bp/s [39] reported for *B*. *subtilis*, are close (note here we compare the total chromosome translocation speeds rather than speeds per chromosome arm). Slightly smaller measured speeds in *B*. *subtilis* may be related to use of SYTOX-Green in earlier measurements as this label does not allow full quantification of DNA movement. Altogether our results indicate that even though *E*. *coli* does not sporulate, its DNA pump FtsK appears to be fully capable of supporting DNA movements to the same extent that occurs during *B*. *subtilis* sporulation. Consistent with the capability to transport large chromosomal regions is the distribution of FtsK loading sites, the KOPS sequences. These sequences are not only present at the replication terminus region but spread throughout the *E*. *coli* chromosome (about one motif every 12–13 kb) [23].

### Translocation timing

The previous research based on chromosome dimer resolution has provided indirect evidence that FtsK activity occurs at late stages of cytokinesis [42, 45] even though FtsK hexamers can be found at the Z-ring before constriction starts [46]. Fluorescence microscopy has also provided evidence of SpoIIIE activity occurring in sporulating *B*. *subtilis* in the late stages of septal closure [47]. Here, we have been able to directly measure when the FtsK mediated pumping starts and stops. We find that on average the translocation starts 8 min before ZipA-GFP label dissociates from the septum although there is a significant variation of this time in a cell population. Dissociation of divisome components has been shown to be an ordered process following the time sequence (FtsZ, ZapA)→(ZipA,FtsA)→(FtsL, FtsQ)→FtsN [48]. The timing of FtsK dissociation has not yet been determined relative to all of these components but it is known to occur after FtsZ leaves [49]. FRAP experiments have shown that ZipA and FtsA dissociate from the septum at the time when the inner membrane effectively closes for diffusion, while FtsZ and ZapA leave before this closure [50]. This finding is consistent with our measurements where we observe that the HupA-mCherry flux through septum stops at the time when ZipA-GFP dissociates from the divisome. At this stage the septal pore has a diameter much smaller than the resolution limit of the microscope (~250 nm).

**Fig 9:**
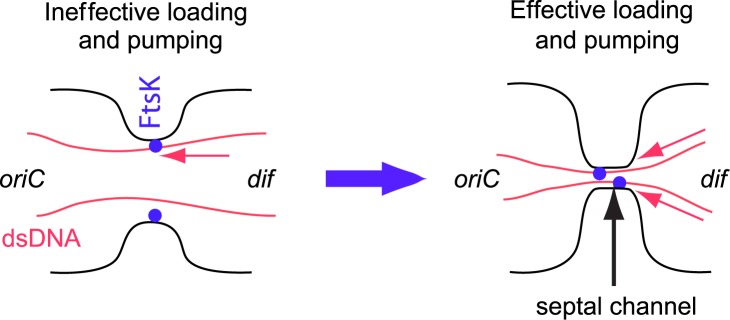
Model relating translocation efficiency and timing. Left: Although FtsK is recruited to Z-ring early, DNA translocation starts when the septum is closing because only then does DNA from the nucleoid become accessible to FtsK hexamers. Moreover, for almost open septum the processivity is low because the FtsK pump(s) has a low probability of rebinding to DNA once the two dissociate. The direction of pumping is to the compartment containing *oriC*. Right: In late stages of division constriction forms a small channel which acts as a diffusion barrier to proteins. The channel does not close before DNA is cleared from it. The processivity of DNA transport in this channel is high because FtsK and DNA remain in close proximity. When the two dissociate from each other then they quickly rebind. Also, FtsK may not be able to reverse its direction when confined by the channel.

To allow DNA movement, a small septal channel must remain in cells whose translocation has not completed at the time when the ZipA-GFP label dissociates from the septum. In some cells such a channel appears to persist for as long as 40 min. A hint at what such a channel may look like is provided by EM images of *ftsK1*::*cat* cells, which have an insertion in the FtsK C-terminal domain. These EM images show the mutant cells forming long septate chains [43] with an approximately 50 nm long and 80 nm wide channel-like connection between daughters. Our measurements imply that such connections are present also in FtsK^+^ cells when translocation occurs after dissociation of ZipA-GFP (Fig 9, bottom). Only after all DNA is cleared from the connection between the daughters can the channel close and the final cell separation take place. DNA in the septal channel appears thus to be a cell cycle checkpoint that prevents separation of daughters as hypothesized before [21].

We find that distribution of translocation start times peaks at about the time of septum closure. Does this finding imply the presence of some biochemical signal that activates FtsK when FtsZ, ZipA, FtsA and ZapA dissociate from the septum? Findings from *in vitro* studies and our measurements argue against this idea. Indeed, in *in vitro* experiments no activation of FtsK was needed [24–26, 28]. These experiments still leave open the possibility that activation may be needed for full length FtsK since in all *in vitro* studies truncated FtsK was used that lacked its N-terminal domain and most of its unstructured linker domain. From our measurements the argument against the presence of a dedicated signal activating full-length FtsK stems from broad distribution of translocation start times (Fig 7A). In many cells translocation clearly started before early Z-ring components dissociated from the septum. These two findings together seem to rule out that there is a biochemical signal that activates FtsK. We therefore propose a simpler explanation that FtsK, which is anchored to membrane at the septum, has only limited access to DNA when the septum has not undergone significant constriction (Fig 9). As the constriction closes, FtsK can reach DNA strands trapped under the septum and load on it. The distribution of normalized Z-ring widths (Fig 8A) further supports this idea showing that translocation does not start when the septum is almost open but it likelihood increases as the septum closes.

### Directionality of DNA movement

Why is DNA sometimes pumped into, and sometimes out from, the smaller daughter compartment? As it is well-established, the directionality of DNA pumping is provided by KOPS sequences [23]. FtsK loads on DNA preferentially at KOPS sequences in an orientation that directs it towards *dif* site at the replication terminus region. KOPS sequences guide FtsK towards *dif* on both replichores and their orientation flips at the replication origin (*oriC*). Altogether, such orientation of KOPS ensures that FtsK moves DNA into the compartment containing *oriC* [38]. In slow growing cells replication origin is positioned in the center of the nucleoid at the time of cell division [51, 52]. If the division plane is positioned closer to the pole than the location of the replication origin then DNA is pumped out from the smaller daughter compartment. Conversely, if more than half of the nucleoid is initially in the smaller daughter compartment then it likely also contains *oriC* and the remaining chromosome is pumped into the smaller daughter. While the above explanation applies to most of the cells, *oriC* position deviates from the nucleoid center in some cells, and in these cells more than 50% of genome can move across the division plane as it is observed in measurements here. However, in the majority of cases our measurements show that DNA tends to be pumped to the compartment where the majority of the chromosome is already present in accordance with the above explanation.

### Comparison to *in vitro* measurements

*In vitro* experiments have shown that FtsK frequently reverses its direction either spontaneously or due to DNA binding proteins [26]. The spontaneous reversals *in vitro* experiments appear to occur on average after few seconds corresponding to translocation of about 10–15 kb of DNA [26, 27]. Due to these reversals *in vitro* measurements tend not to show net DNA movement in longer time scales. Reversals and short unbinding periods of FtsK from DNA likely go unnoticed in our measurements, which probe DNA movement on time scales of a few minutes. However, in these longer time-scales we observe uninterrupted progression of translocation in the majority of cases. In only about 10% of cases, pauses in translocation of 8 min or longer are present. In no cases did we observe reversal of translocation direction. The high processivity at these longer time scales comes despite the high likelihood of FtsK colliding with the transcription machinery head on or by overtaking it by speed (transcription progresses at speeds of <100 bp/s). The high observed processivity *in vivo* may be related to the finding that translocation occurs very late in the cell cycle when the septum has closed to a narrow channel. In such channels, reversals may not be possible. Also, after dissociation FtsK may be able to quickly rebind to DNA *in vivo* while during *in vitro* experiments, truncated FtsK will likely diffuse away. Septa that are nearly closed may also prevent DNA diffusing back into the daughter compartment from where it was pumped out. Narrow constrictions thus may be required, not only for effective loading, but also for unidirectional and continuous DNA movement from one daughter compartment to another (Fig 9). This idea is consistent with the finding that the majority of DNA movement occurs once the septum is closed to small channel having diameter less than 250 nm and that translocation speeds tend to increase the later the process occurs in cell cycle.

To conclude, our work demonstrates that *E*. *coli* cells are capable of repositioning their chromosomes when their cell division plane is not properly localized. This large scale repositioning is mediated by FtsK, which is capable of translocating as much as 80% of chromosome at an average rate of 1700 bp/s. Most of this translocation occurs after early arriving Z-ring proteins have left the constriction and radius of constriction shrank below the resolution limit of our microscope (~250 nm). In some cells earlier pumping is also present indicating that the timing of translocation may be determined by FtsK ability to bind to DNA when the septum is not closed. Our work also implies that DNA located within constriction realizes a cell-cycle checkpoint that prevents daughters from separating from each other. It will be an interesting question for further studies to find if DNA sterically prevents constriction from full closure or if there is some dedicated regulatory mechanism involved.

## Materials and methods

### Strains and growth conditions

All strains used in this study were derivatives of *E*. *coli* K-12 (Keio collection strain BW25113). Strains were constructed using phage P1 mediated transduction, and are described in detail in S1 Text in the Supplemental Material. Deletions in Keio collection [53] that were used for strain construction were verified by PCR using primers flanking the target gene. A substitution of K997A in FtsK was verified by sequencing. All bacteria were grown and imaged in M9 minimal medium (Teknova) supplemented with 2 mM magnesium sulfate and with 0.3% glycerol at 28°C. Kanamycin (20 μg/ml), ampicillin (20 μg/ml) and chloramphenicol (20 μg/ml) were used when required.

### Preparation of cells for microscopy

Bacterial cultures were grown at 28°C with shaking overnight in M9 glycerol. The following day, cultures with an A_600_ between 0.1 and 0.4 were diluted to an A_600_ of 0.02 and grown until A_600_ of 0.08. Cells were then concentrated for imaging. For nucleoid staining, SYTOX-Green solution (Fisher Scientific; final concentration 800 nM) or DAPI (Fisher Scientific; final concentration 5 μg/ml) was added to a culture medium at A_600_ = 0.04 and grown by shaking for 2 hours. When needed Δ*slmA* Δ*minC* Δ*zapB* cells with ZipA-GFP (strain MB16) were induced with 60 μM IPTG for 3.5 hrs before imaging.

For time lapse imaging home-made glass bottom dishes were used. Cells were pipetted onto a #1.5 glass coverslip fitted to the bottom of the dish and covered with about 0.5 cm thick slab of M9-glycerol 2% agarose. No antibiotics were used during imaging. The agarose was supplemented with SYTOX-Green solution (250 nM) for nucleoid imaging but no DAPI was added to the agarose as this addition appeared to be toxic to the cells. 40 μM IPTG was used in agarose for strains with ZipA-GFP.

### Fluorescence microscopy

A Nikon Ti-E inverted fluorescence microscope with a 100X NA 1.40 oil immersion phase contrast objective was used for imaging the bacteria. Fluorescence was excited by a 200W Hg lamp through an ND4 or ND4+ND8 (for DAPI time-lapse) neutral density filter. Chroma 41004, 41001, 31000v2 filter-cubes were used to record mCherry, GFP and DAPI images, respectively. Images were captured by an Andor iXon DU897 camera and recorded using NIS-Elements software.

### Image analysis

Matlab along with the Image Analysis Toolbox and DipImage Toolbox (http://www.diplib.org/) was used for image analysis. In addition to Matlab, simpler image processing such as contrast and brightness adjustments were done using ImageJ software (v1.41o). In all analysis corrections to subpixel shifts between different image planes were applied first. To find normalized intensities, intensity line profiles along the long axes of the cell were extracted. The profiles were averages of 9 pixels wide (≈ 1 μm) lines, which extended beyond cell poles along the long axes of the cell. Intensity profile of the phase contrast image was used to determine the position of the constriction. The position of the constriction divided the intensity profiles of the nucleoid labels (mCherry, DAPI or SYTOX-Green) into two sections corresponding to the smaller and larger daughter cell. After background subtraction, total fluorescent intensities from both sections were calculated along with the normalized intensity (as defined in Fig 1B). To determine width of the Z-ring, first its position along the long axes of the cell was determined from a longitudinal line profile from ZipA-GFP image. Line profile along short axes was then calculated at the location of the Z-ring. From this line profile the width was determined by a Gaussian fitting and by calculating the 2^nd^ normalized moment of the profile.

### Estimating DNA amount from cell length

To determine how the amount of DNA in the cells relates to cell length the cells were fixed in 2% paraformaldehyde for 15 min shaking at 28°C and then permeabilized in 0.1% TritonX-100 for 20 min. After both fixation and permeabilization the cells were washed in 1x PBS. The cells were then stained with DAPI (1 μg/ml) and imaged between two sealed glass slides. For measurements of total fluorescence intensity and cell length, MicrobeTracker software [54] was used. Total intensity distribution from DAPI labelled cells shows clear peak in cells with two fully replicated chromosome. This peak intensity was used to evaluate DNA amount in other cells (SI S5A Fig). Plotting calibrated intensities/DNA amounts as a function of cell length yields relationship *DNA Amount* = 0.92(*L*_*cell*_ − 0.53) (SI S5B Fig). Here, *DNA Amount* is in genome units (4.6 Mb) and cell length, *L*_*cell*_, in micrometers.

## Supporting information

S1 Text Strain construction. (DOCX)

S1 Fig Translocation curves showing pauses. Normalized intensity of one MB16 cell (top) and one JM30 cell (bottom) showing pauses longer than 8 min.
(TIF)

S2 Fig Translocation speed vs. growth rate. Solid line is a linear fit. Pearson correlation coefficient from the fitting is R = 0.066. Data from strains JM30 and MB16 is combined. N = 46.
(TIF)

S3 Fig Comparison of measurements without and with ZipA-GFP label. A, B: Initial vs. final normalized intensity of DAPI label in the smaller daughter’s compartment. (Left column N = 22; Right column, N = 24)
C, D: The total translocated amount into the smaller daughter’s compartment. Positive amounts correspond to DNA movement to and negative amounts from the smaller daughter compartment.
E, F: Distribution of translocation speeds. Mean and standard deviation are indicated.
(TIF)

S4 Fig Quantification of DNA movement in *ΔslmA ΔminC ΔzapB* cells during translocation assuming that chromosomes are replicating at the time of translocation. Determination of DNA amount from cell length is described in Experimental Methods section in the Main Text.
A: Estimated amount of DNA in the smaller daughter compartment in the beginning and end of translocation. The amount of DNA is given in genome units (4.6 Mb). Dashed horizontal lines correspond to integer genome equivalents. Solid diagonal line corresponds to no change in DNA amount during the division.
B: Distribution of DNA amount that crossed the division plane during translocation. DNA amounts are given in genome units. Positive amounts correspond to DNA moving into the smaller daughter compartment and negative amounts out from it.
C: Distribution of translocation speeds. The average translocation speed 2100±800 bp/s. Data from strains JM30 and MB16 is combined. N = 46.
(TIF)

S5 Fig Quantification of DNA movement during translocation in *ΔslmA ΔminC ΔzapB ftsKK997A* cells. DNA amount is expected to be integer number of genome equivalents at the time of division.
A: Estimated amount of DNA in the smaller daughter compartment in the beginning and end of translocation. Dashed horizontal lines correspond to integer genome equivalents. Solid diagonal line corresponds to no change in DNA amount during the division. N = 13.
B: Distribution of DNA amount that crossed the division plane during translocation. Positive amounts correspond to DNA moving into the smaller daughter compartment and negative amounts out from it.
(TIFF)

S6 Fig Relationship between DNA amount and cell length in *ΔslmA ΔminC ΔzapB* cells. A: Distribution of total fluorescent intensities from DAPI labelled cells. Prior to DAPI staining the cells have been fixed and permeabilized. See Materials and Methods section in the Main Text for additional experimental details. The peak corresponding to two fully replicated chromosomes is marked. Strain MB16 (without induction). N = 321.
B: Based on intensity of the two chromosome peak, the DNA amounts in these cells are calibrated and plotted against cell length. Solid line shows a fitting line to these data describing the relationship
DNA Amount = 0.92(L_cell_-0.53); (R = 0.93).
(TIF)

S1 Movie DNA movement during division in asymmetrically dividing *ΔslmA ΔminC ΔzapB* cell that is shown in Fig 1 in the Main Text. Fluorescent image of HupA-mCherry is overlaid with phase contrast image of the cell. Scale bar corresponds to 2 μm.
(AVI)

S2 Movie Further growth and division of daughters from the cell that is shown in Fig 1 in the Main Text. Scale bar corresponds to 2 μm.
(AVI)

S3 Movie DNA movement during division in asymmetrically dividing *ΔslmA ΔminC ΔzapB* cell that is shown in Fig 3 in the Main Text. Nucleoid is labelled with DAPI (top panel) and HupA-mCherry (bottom). Scale bar corresponds to 2 μm.
(AVI)

S4 Movie Growth, division and lysis in a small colony of *ΔslmA ΔminC ΔzapB ftsK K997A* cells. Fluorescent image of HupA-mCherry is overlaid with phase contrast image. Scale bar corresponds to 2 μm.
(AVI)

S5 Movie DNA movement during division in asymmetrically dividing *ΔslmA ΔminC ΔzapB ftsK K997A* cell that is shown in Fig 4 in the Main Text. Nucleoid is labelled with DAPI (top panel) and HupA-mCherry (bottom). Scale bar corresponds to 2 μm.
(AVI)

S1 Dataset All translocation traces accompanying Fig 1. (PDF)

S2 Dataset All translocation traces accompanying Fig 2. (PDF)

S3 Dataset All translocation traces from strain JM30 accompanying Figs 3 and 5. (PDF)

S4 Dataset All translocation traces from strain MB16 accompanying Figs 3, 5, 7 and 8. (PDF)

S5 Dataset All translocation traces accompanying Fig 4. (PDF)
