# Supplementary material for: Kinetics of large-scale chromosomal movement during asymmetric cell division in Escherichia coli
Source: PLoS Genet. 2017 Feb 24;13(2):e1006638. doi: 10.1371/journal.pgen.1006638 (PMC5345879; doi:10.1371/journal.pgen.1006638)
Supplement: S1 Text — (DOCX) [file pgen.1006638.s001.docx]

**Strain construction**

**Strain MB16** (Δ*minC*::*frt,* Δ*slmA*::*frt,* Δ*zapB*::*frt,* Δ*zipA*::*P_lac_-zipA-gfp-amp,* Δ*hupA::hupA-mCherry-frt-kan-frt*) was constructed by sequential P1 transductions. Keio collection parental strain BW25113 (CGSC# 7636) was P1 transduced with a lysate from strain JW1165 (Δ*minC*:: *frt-kan-frt)*, JW5641-1 (Δ*slmA*:: *frt-kan-frt),* and JW3899-1 *(*Δ*zapB*:: *frt-kan-frt)* to introduce respective deletions (Baba et al., 2006). After every P1 transduction, the kanamycin resistance cassette flanked by the Flp recognition target from the gene deletion site was removed using the pCP20 plasmid (Cherepanov and Wackernagel, 1995). The resulting triple deletion strain was P1 transduced with a lysate from strain TB86 (λCH151) (a kind from P. de Boer from Case Western Reserve University; Bernhard and de Boer, 2005) to introduce the inducible ZipA-GFP. Finally, the resultant strain employed as a recipient for P1 transduction using a lysate from strain PB384 (a kind gift from P. Bissichia and D. Sherratt from Oxford University) to introduce HupA-mCherry. Desired deletions were confirmed by PCR analysis.

**Strain JM30** (Δ*minC*::*frt,* Δ*slmA*::*frt,* Δ*zapB*::*frt,* Δ*hupA::hupA-mCherry-frt-kan-frt*) was constructed by sequential P1 transductions of Keio collection parental strain BW25113. The triple deletion strain was P1 transduced with a lysate from strain PB384 (a kind gift from P. Bissichia and D. Sherratt) to introduce HupA-mCherry.

**Strain JM38** (Δ*minC*::*frt,* Δ*slmA*::*frt,* Δ*zapB*::*frt,* Δ*hupA::hupA-mCherry-frt-kan-frt,* Δ*ftsK::ftsK^ATP-^ -cm*), is a triple deletion mutant containing a substitution K997A in Walker A motif of FtsK. The strain was constructed by P1 transduction using strain FC1 (a kind gift from F. X. Barre from CNRS, France; Kennedy et al., 2008) as a donor strain, selecting for chloramphenicol resistance. Presence of substitution K997A in FtsK was confirmed by sequencing.

Baba, T., Ara, T., Hasegawa M., Takai, Y., Okumura, Y., Baba, M., Datsenko, K.A., Tomita, M., Wanner, B.L., Mori, H. 2006. Construction of Escherichia coli K-12 in-frame, single-gene knockout mutants: the Keio collection. *Mol Syst Biol* 2:1-11

Bernhardt, T.G., de Boer, P.A. 2005. SlmA, a nucleoid-associated, FtsZ binding protein required for blocking septal ring assembly over Chromosomes in *E. coli. Molecular Cell* 18(5):555-64.

Cherepanov, P.P., Wackernagel, W. 1995. Gene disruption in Escherichia coli: TcR and KmR cassettes with the option of Flp-catalyzed excision of the antibiotic-resistance determinant. *Gene* 158(1):9-14.

Kennedy, S.P., Chevalier, F. and Barre, F.-X. (2008) Delayed activation of Xer recombination at *dif* by FtsK during septum assembly in *Escherichia coli*. *Mol. Microbiol.* 68: 1018-1028.
