# Supplementary figures and images for: Kinetics of large-scale chromosomal movement during asymmetric cell division in Escherichia coli
Source: PLoS Genet. 2017 Feb 24;13(2):e1006638. doi: 10.1371/journal.pgen.1006638 (PMC5345879; doi:10.1371/journal.pgen.1006638)

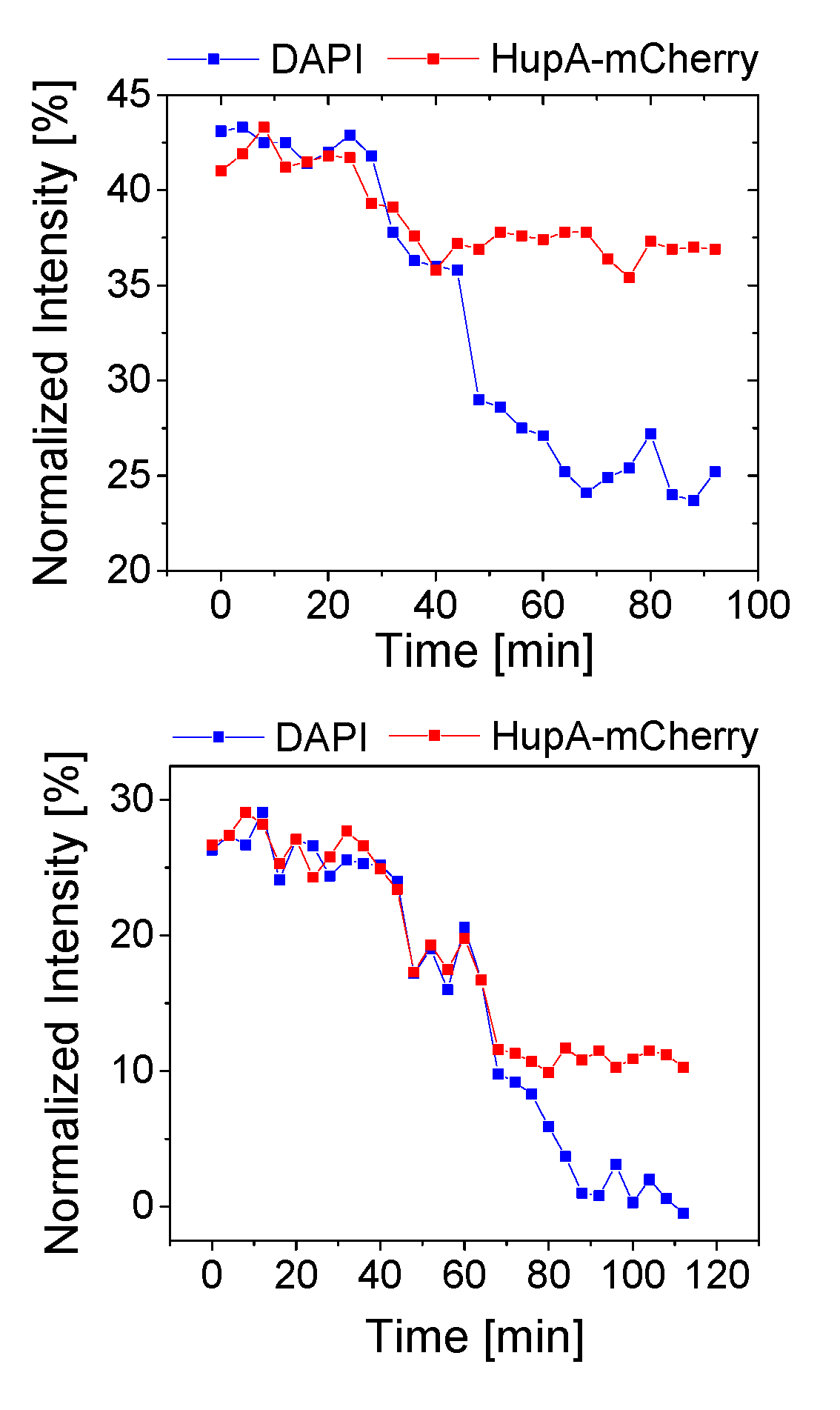

Supplement: S1 Fig — Normalized intensity of one MB16 cell (top) and one JM30 cell (bottom) showing pauses longer than 8 min. (TIF) [file pgen.1006638.s002.tif]

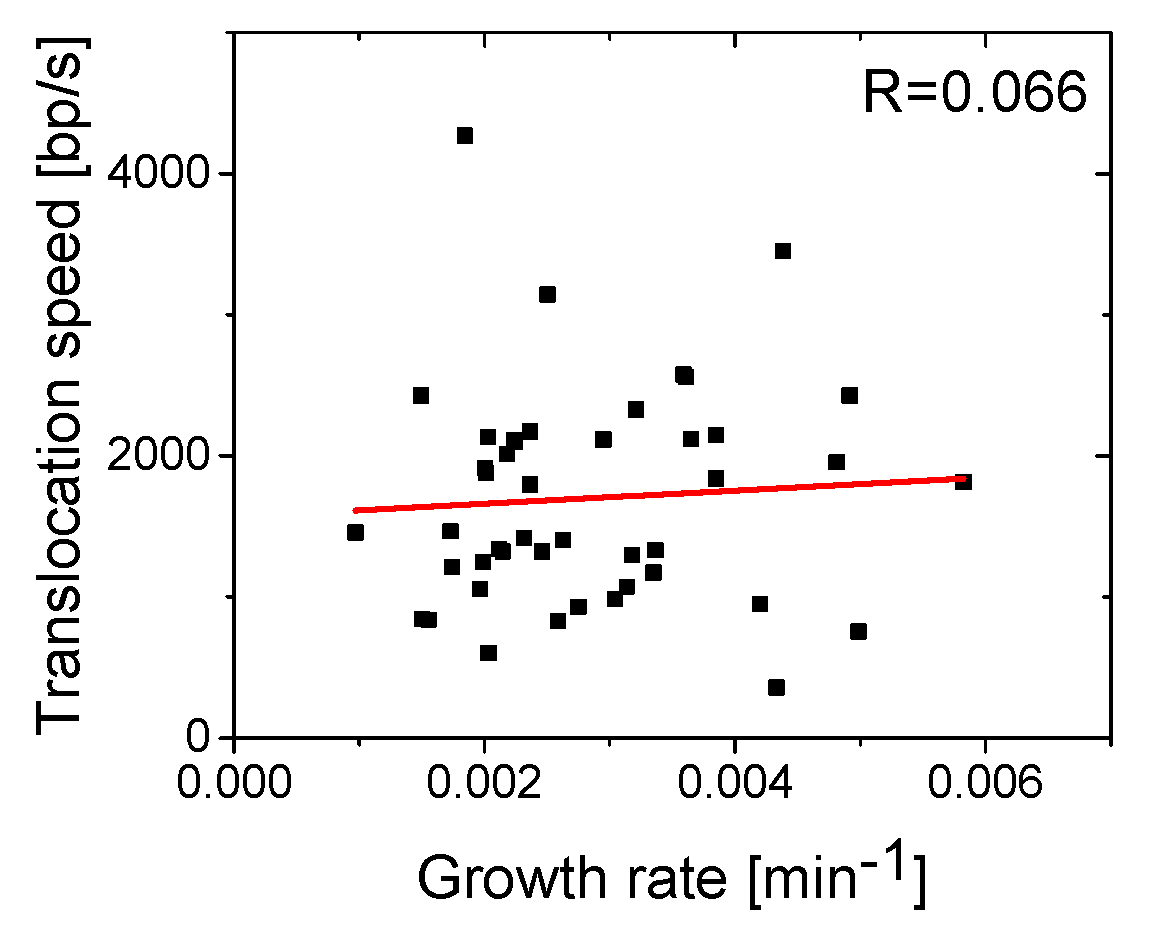

Supplement: S2 Fig — Solid line is a linear fit. Pearson correlation coefficient from the fitting is R = 0.066. Data from strains JM30 and MB16 is combined. N = 46. (TIF) [file pgen.1006638.s003.tif]

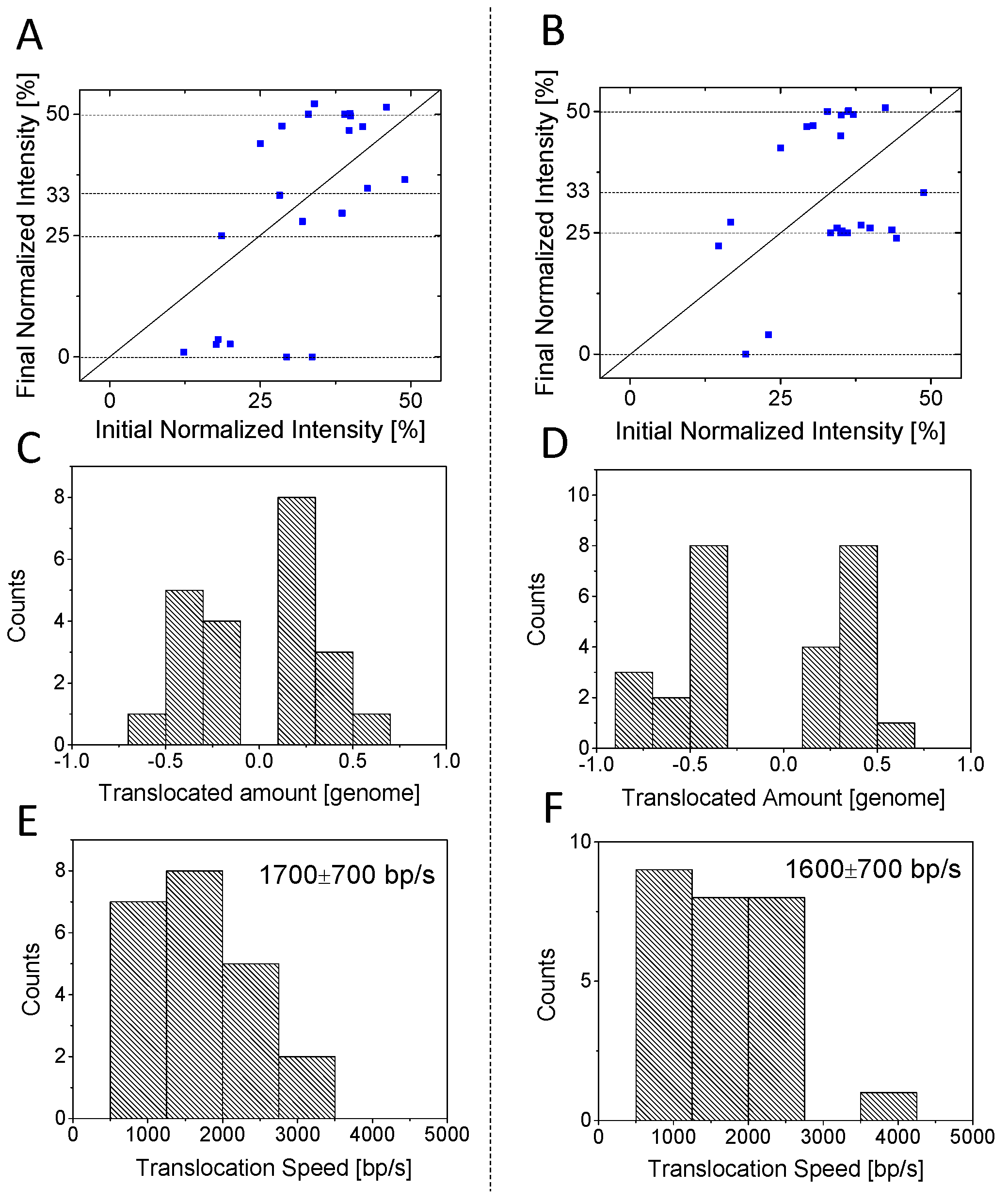

Supplement: S3 Fig — A, B: Initial vs. final normalized intensity of DAPI label in the smaller daughter’s compartment. (Left column N = 22; Right column, N = 24) C, D: The total translocated amount into the smaller daughter’s compartment. Positive amounts correspond to DNA movement to and negative amounts from the smaller daughter compartment. E, F: Distribution of translocation speeds. Mean and standard deviation are indicated. (TIF) [file pgen.1006638.s004.tif]

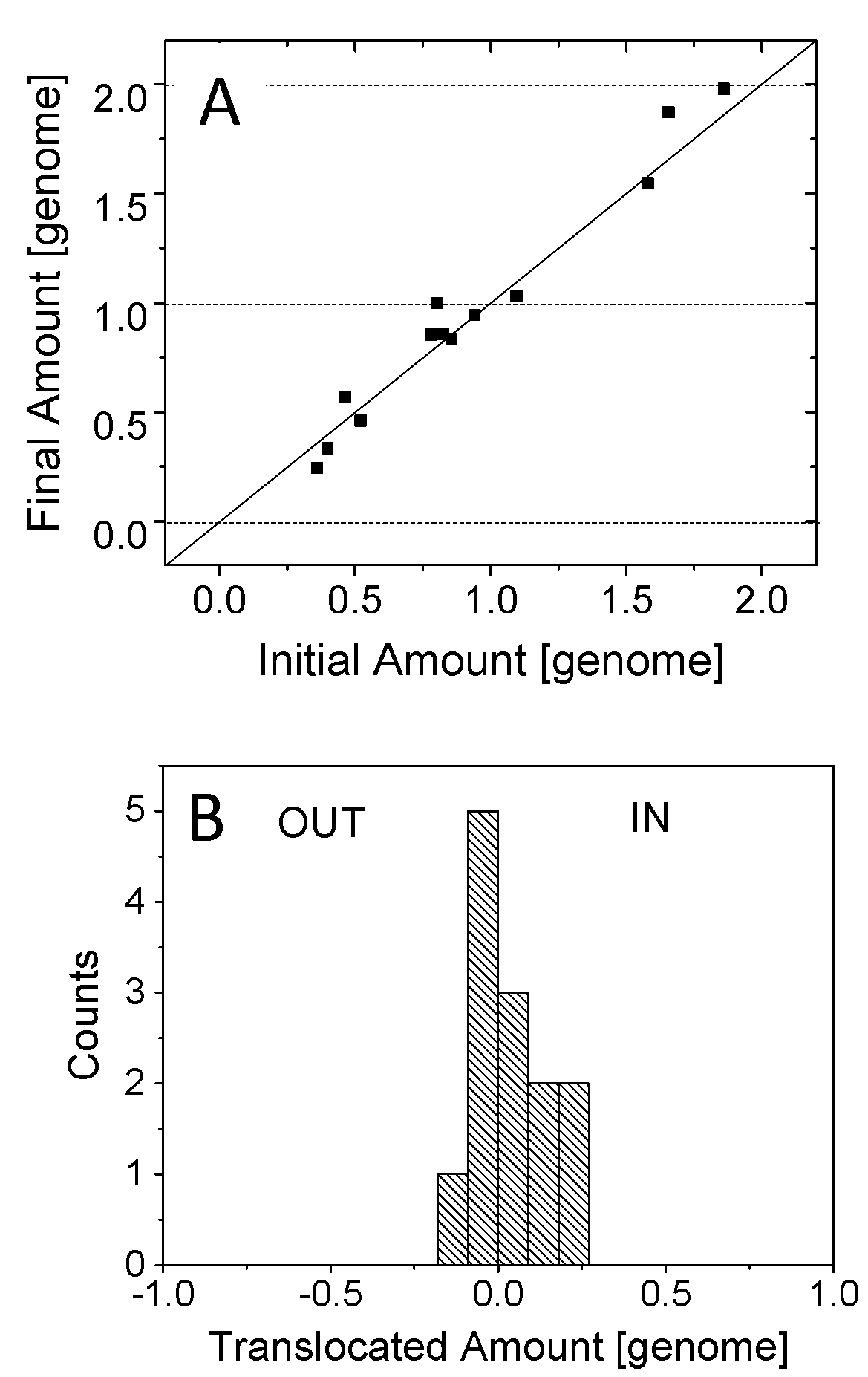

Supplement: S5 Fig — DNA amount is expected to be integer number of genome equivalents at the time of division. A: Estimated amount of DNA in the smaller daughter compartment in the beginning and end of translocation. Dashed horizontal lines correspond to integer genome equivalents. Solid diagonal line corresponds to no change in DNA amount during the division. N = 13. B: Distribution of DNA amount that crossed the division plane during translocation. Positive amounts correspond to DNA moving into the smaller daughter compartment and negative amounts out from it. (TIFF) [file pgen.1006638.s006.tiff]

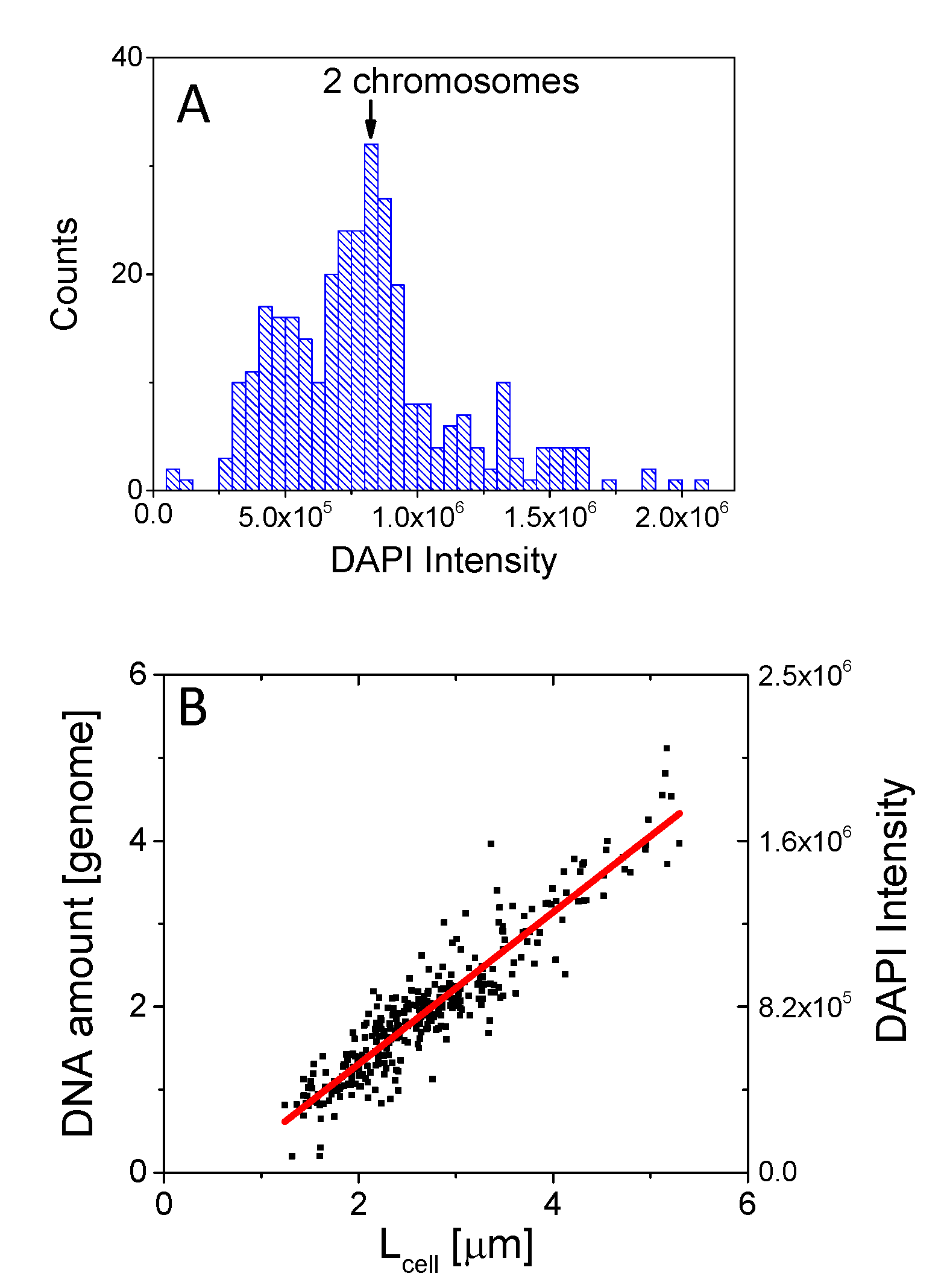

Supplement: S6 Fig — A: Distribution of total fluorescent intensities from DAPI labelled cells. Prior to DAPI staining the cells have been fixed and permeabilized. See Materials and Methods section in the Main Text for additional experimental details. The peak corresponding to two fully replicated chromosomes is marked. Strain MB16 (without induction). N = 321. B: Based on intensity of the two chromosome peak, the DNA amounts in these cells are calibrated and plotted against cell length. Solid line shows a fitting line to these data describing the relationship DNA Amount = 0.92(Lcell-0.53); (R = 0.93). (TIF) [file pgen.1006638.s007.tif]
