# Supplementary material for: Kinetics of large-scale chromosomal movement during asymmetric cell division in Escherichia coli
Source: PLoS Genet. 2017 Feb 24;13(2):e1006638. doi: 10.1371/journal.pgen.1006638 (PMC5345879; doi:10.1371/journal.pgen.1006638)

Strain JM30

HupA-mCherry fluorescence

IpeakRatio=(Normalized Intensity)/100

Frame time = 4 min

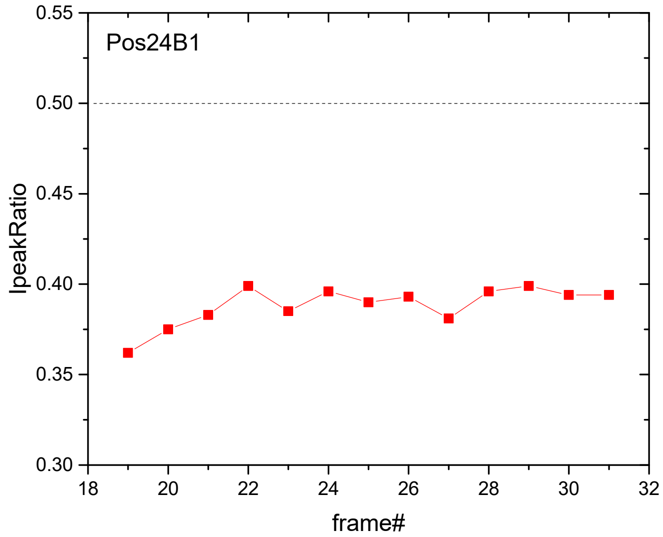

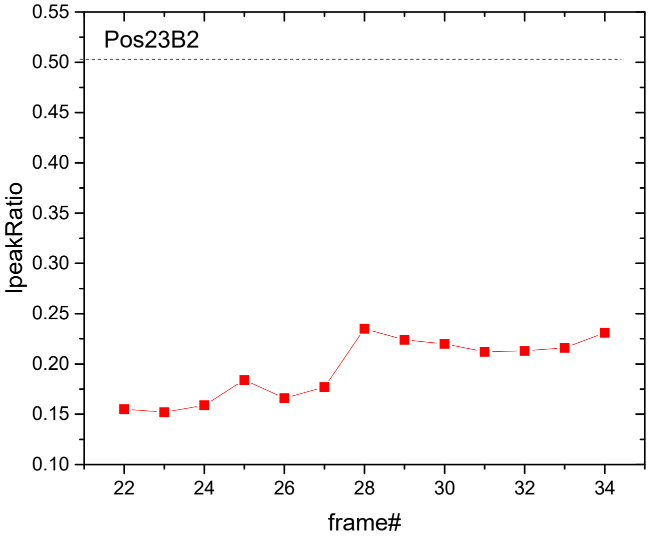

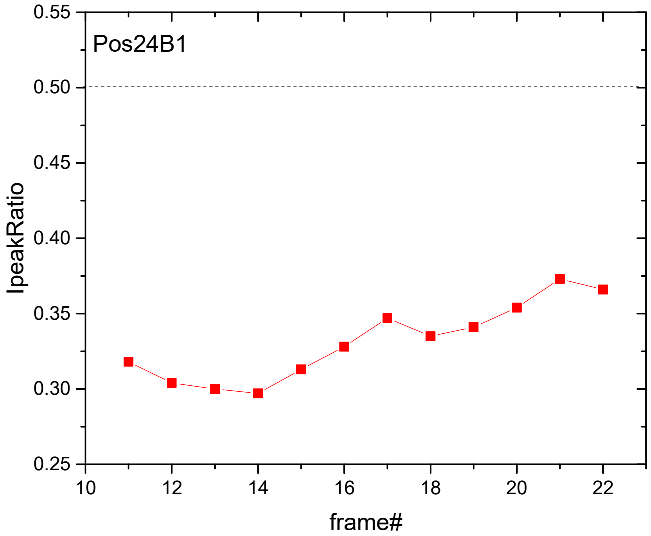

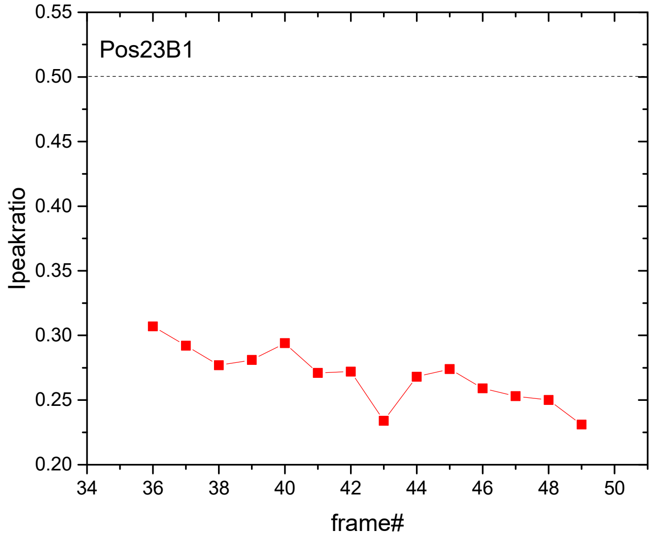

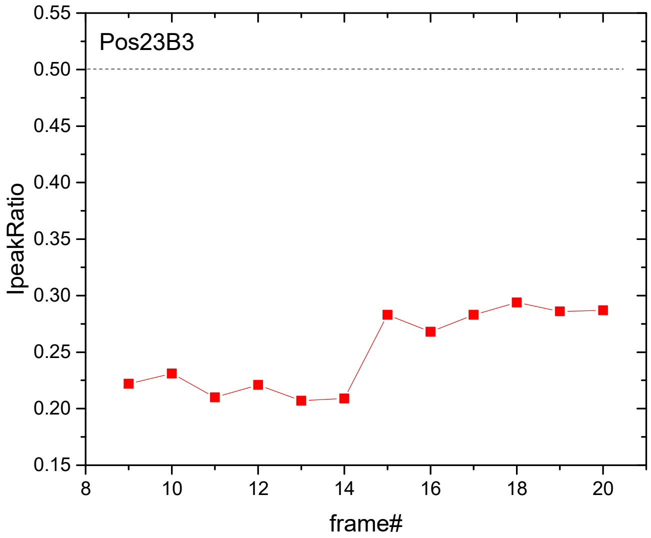

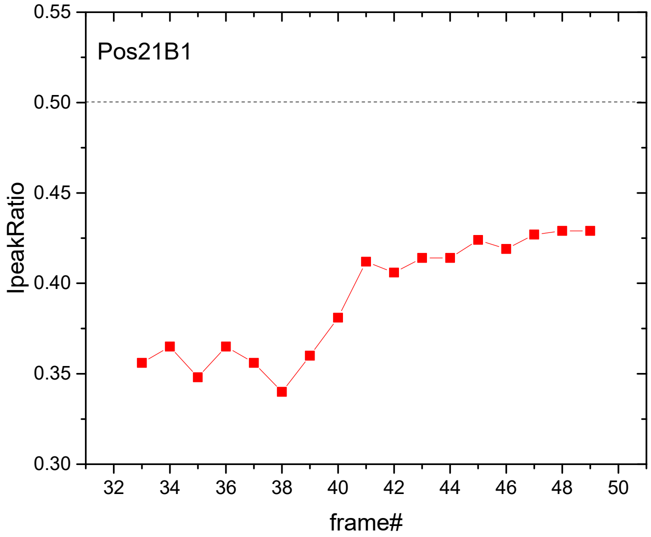

Strain JM30

HupA-mCherry fluorescence

IpeakRatio=(Normalized Intensity)/100

Frame time = 4 min

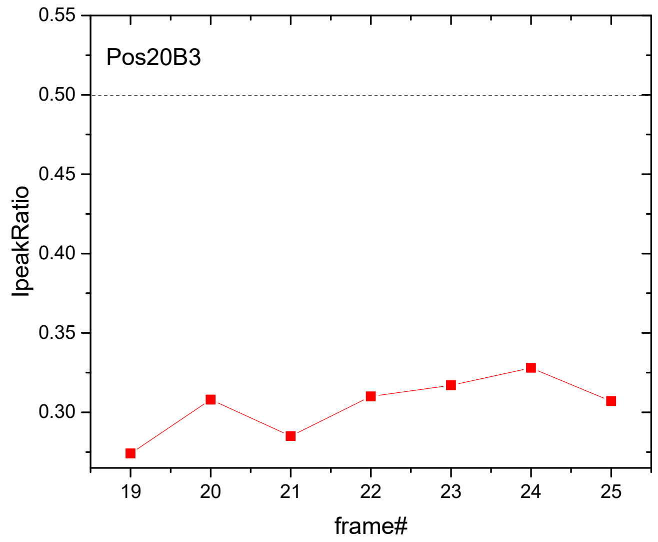

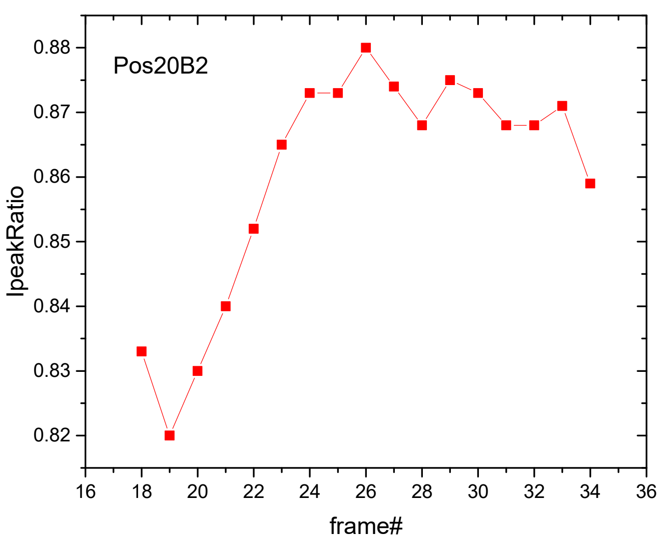

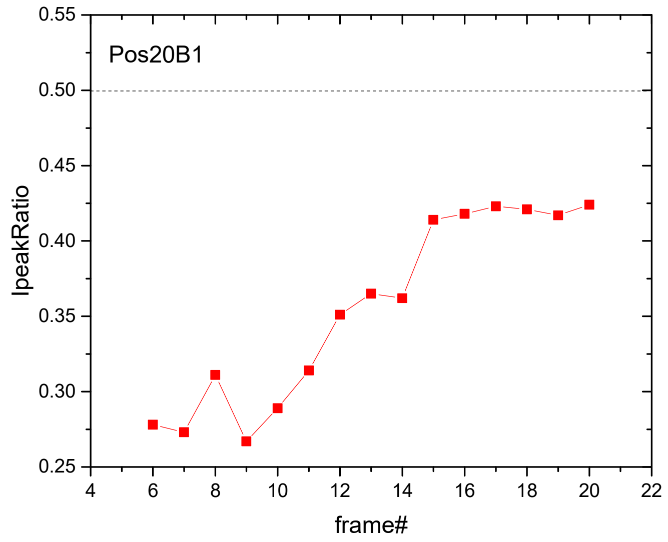

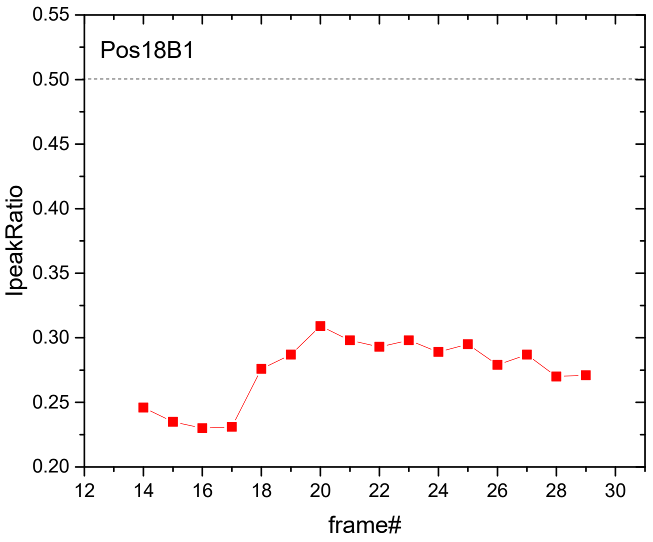

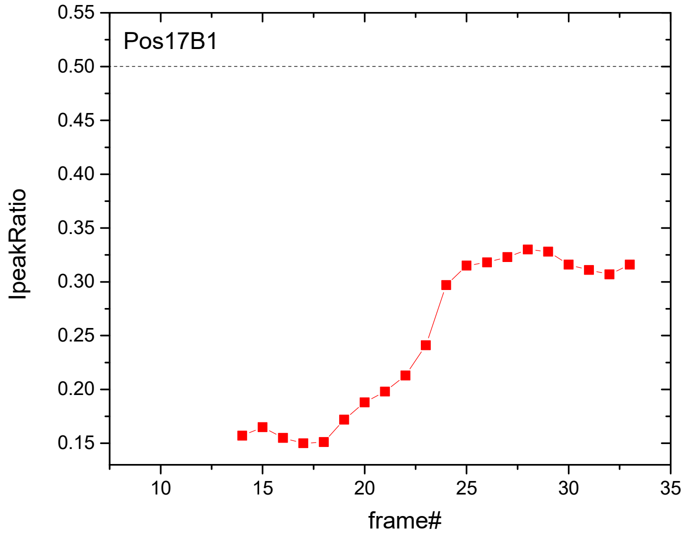

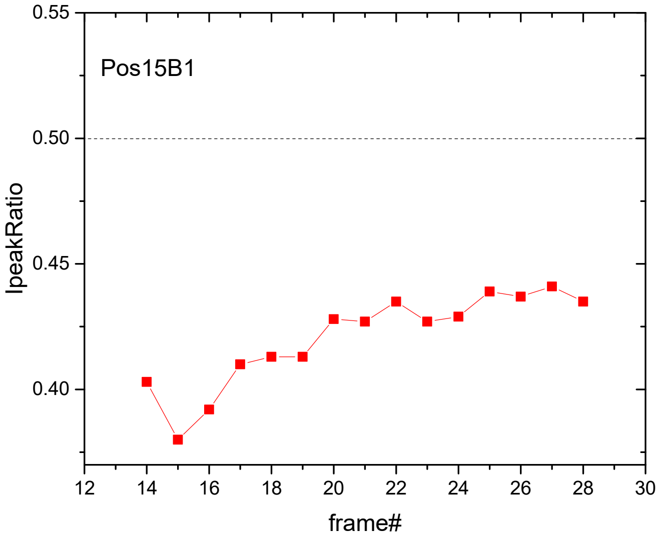

Strain JM30

HupA-mCherry fluorescence

IpeakRatio=(Normalized Intensity)/100

Frame time = 4 min

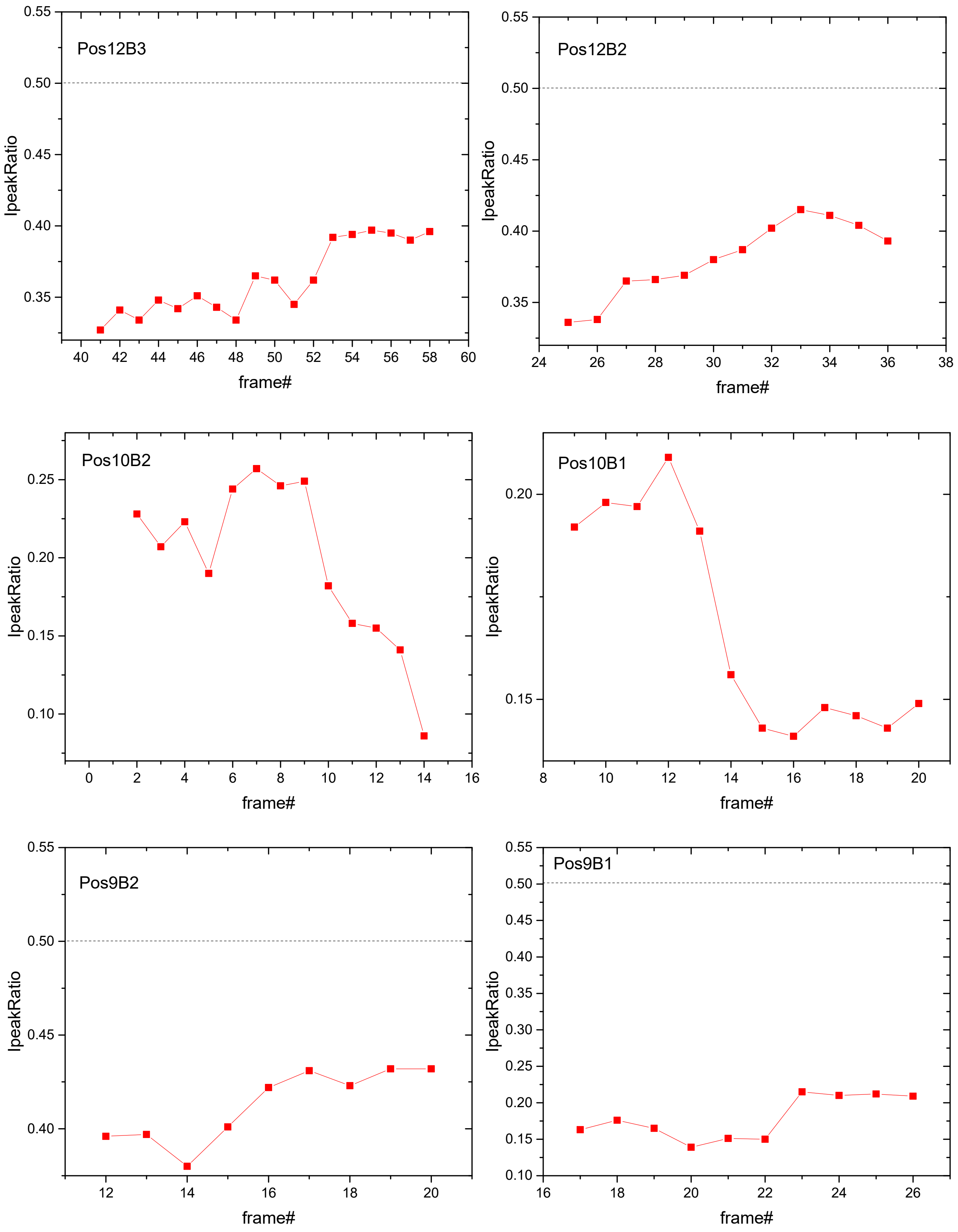

Strain JM30

HupA-mCherry fluorescence

IpeakRatio=(Normalized Intensity)/100

Frame time = 4 min

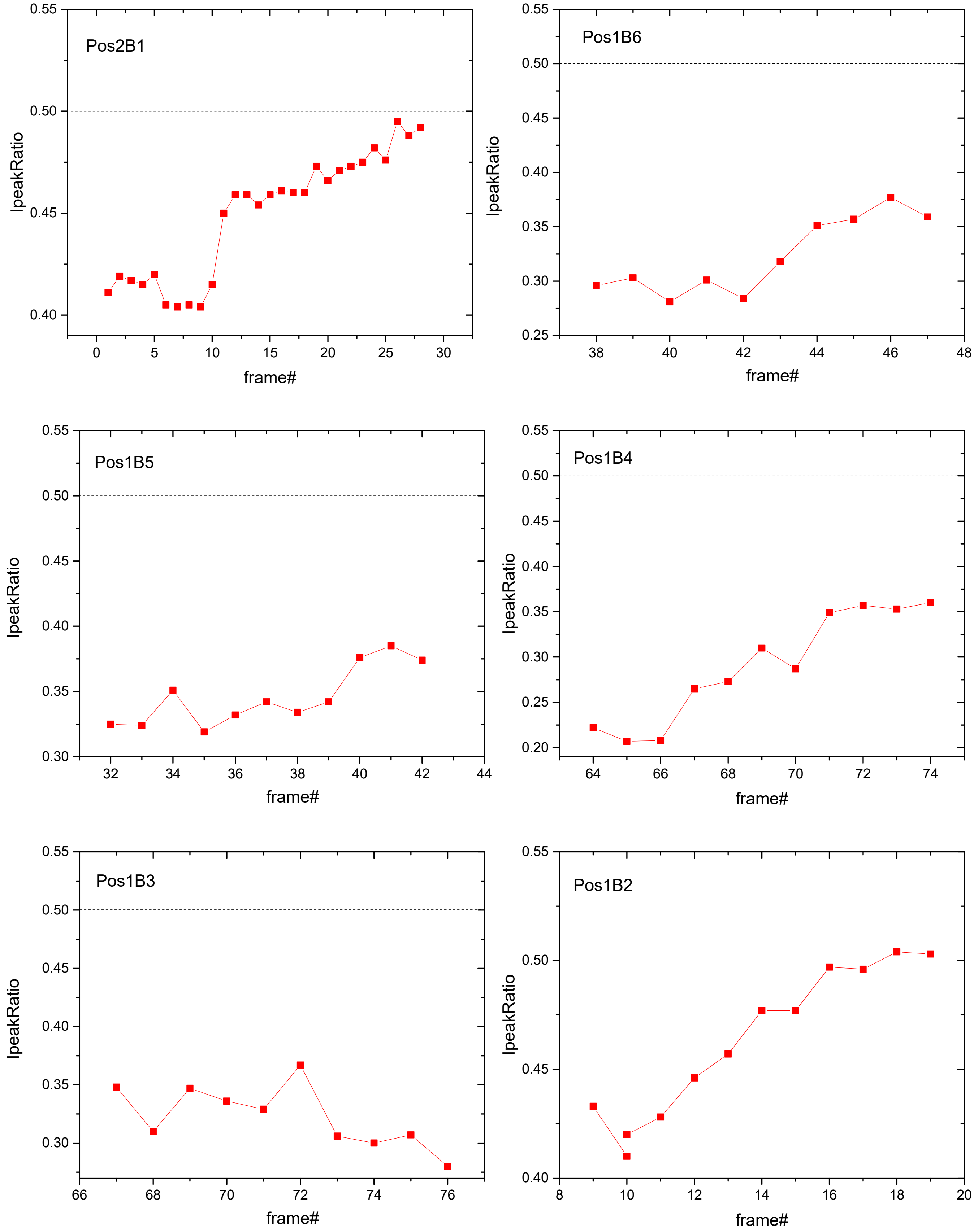

Strain JM30

HupA-mCherry fluorescence

IpeakRatio=(Normalized Intensity)/100

Frame time = 4 min

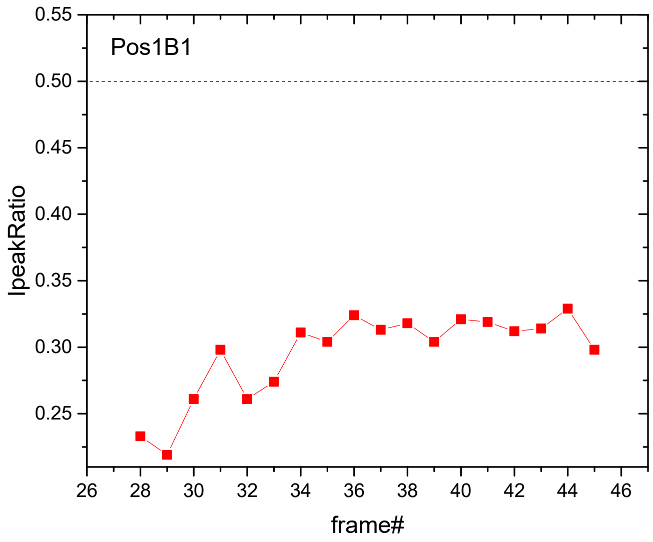

Supplement: S1 Dataset — (PDF) [file pgen.1006638.s013.pdf]
