# Supplementary material for: Kinetics of large-scale chromosomal movement during asymmetric cell division in Escherichia coli
Source: PLoS Genet. 2017 Feb 24;13(2):e1006638. doi: 10.1371/journal.pgen.1006638 (PMC5345879; doi:10.1371/journal.pgen.1006638)

Strain MB16

HupA-mCherry and DAPI fluorescence; ZipA-GFP induced

IpeakRatio=(Normalized Intensity)/100

0 min. corresponds to time when ZipA-GFP label dissociates from the septum

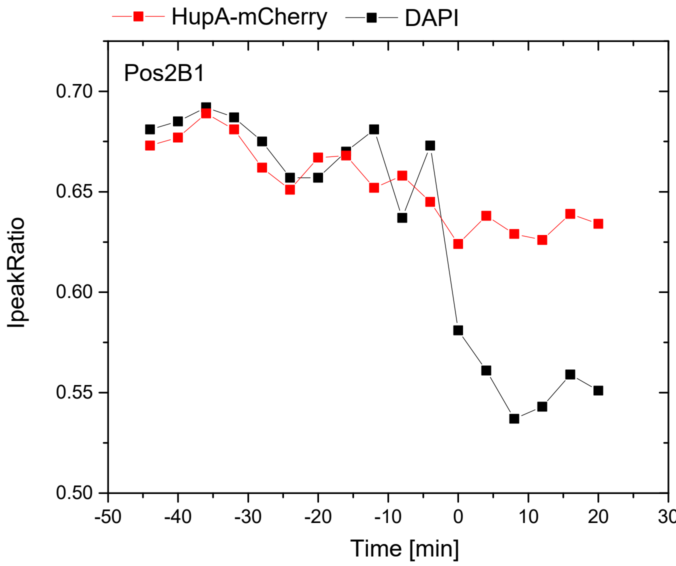

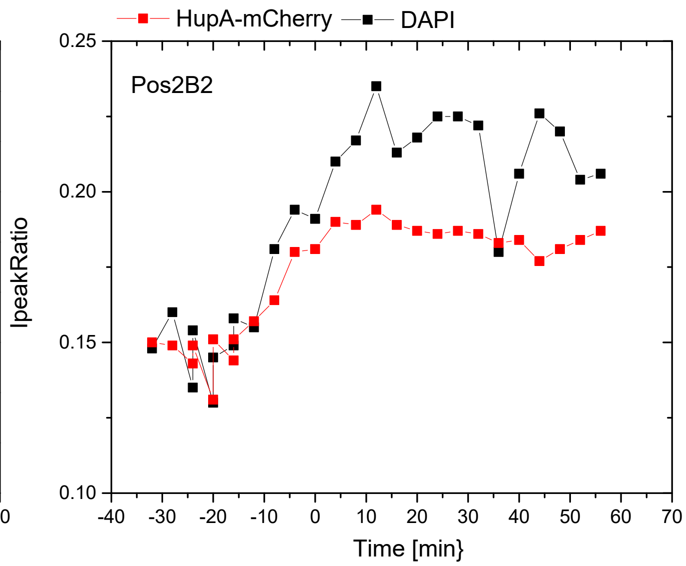

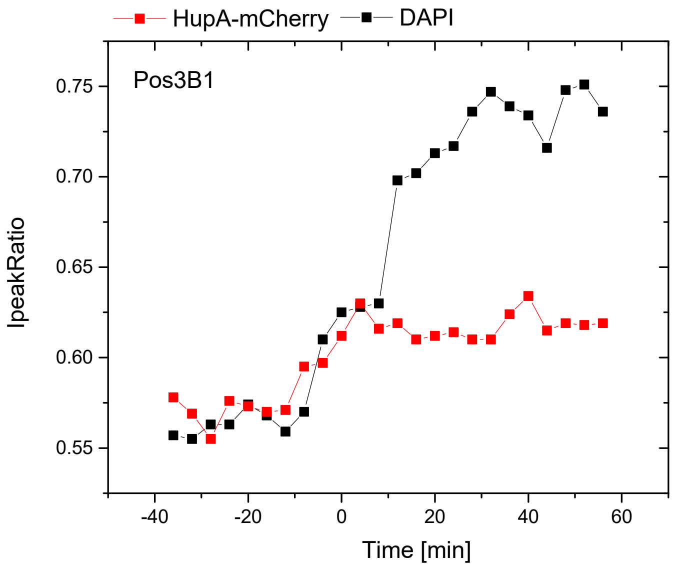

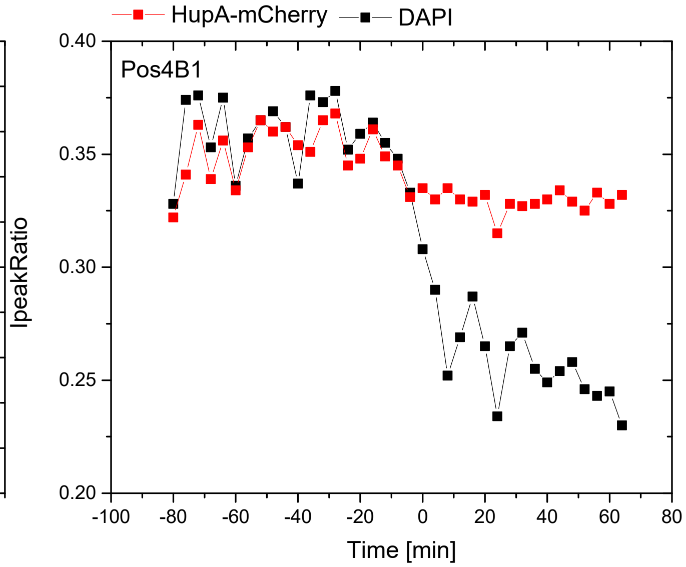

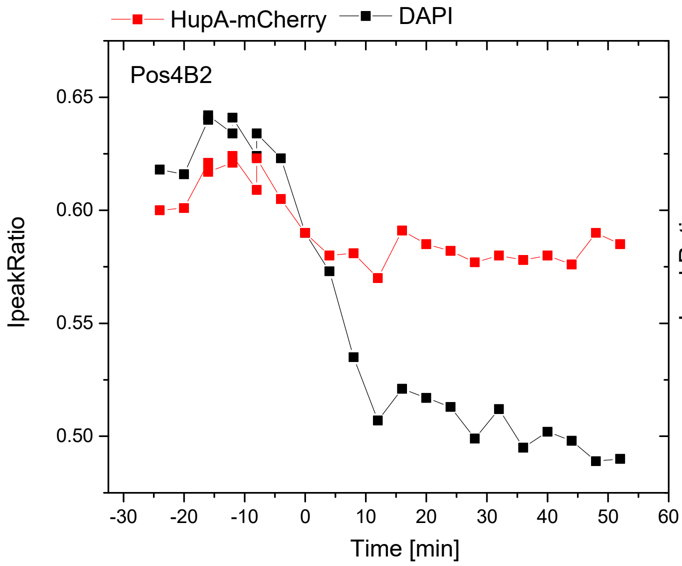

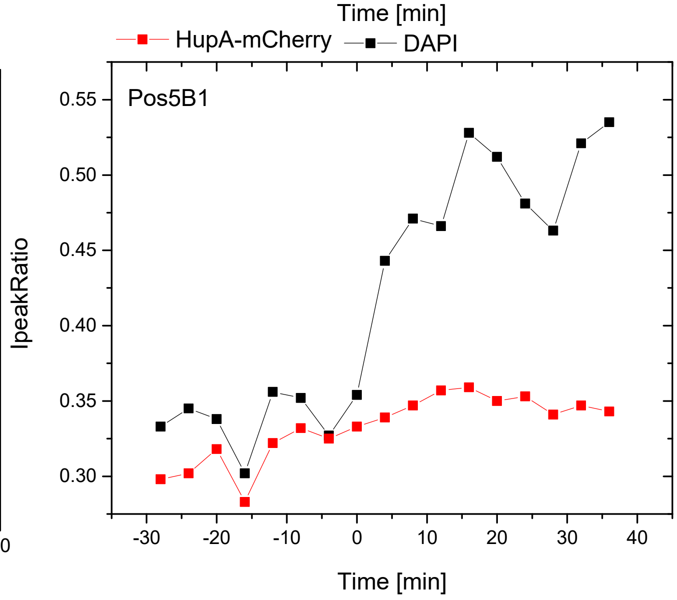

Strain MB16

HupA-mCherry and DAPI fluorescence; ZipA-GFP induced

IpeakRatio=(Normalized Intensity)/100

0 min. corresponds to time when ZipA-GFP label dissociates from the septum

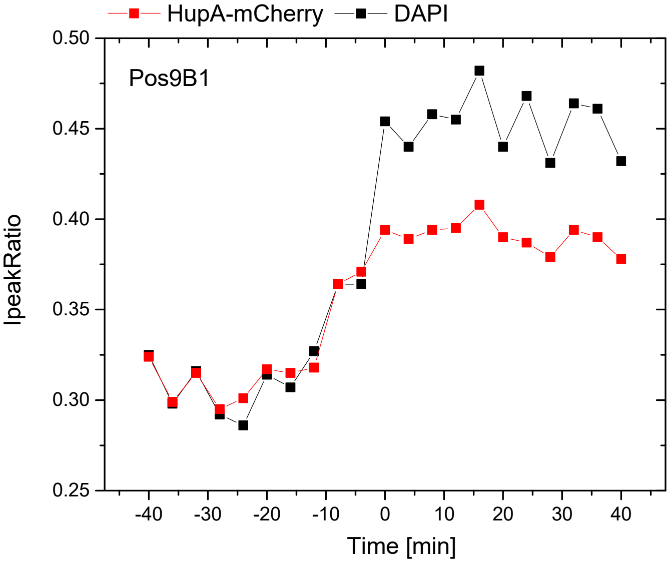

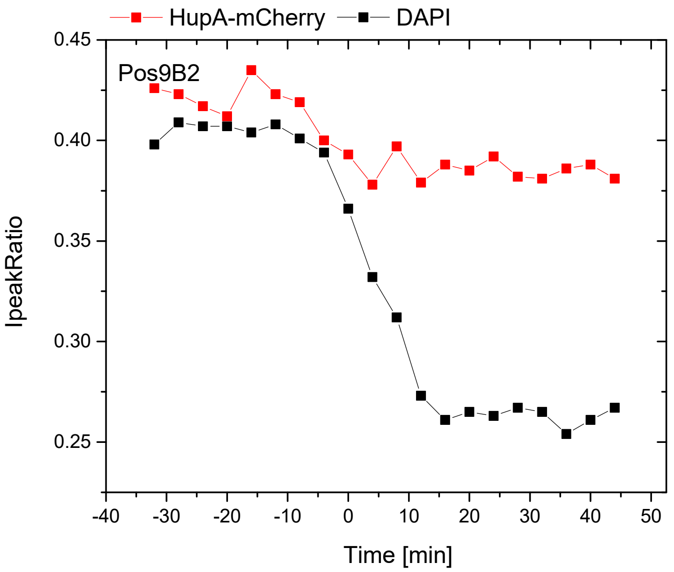

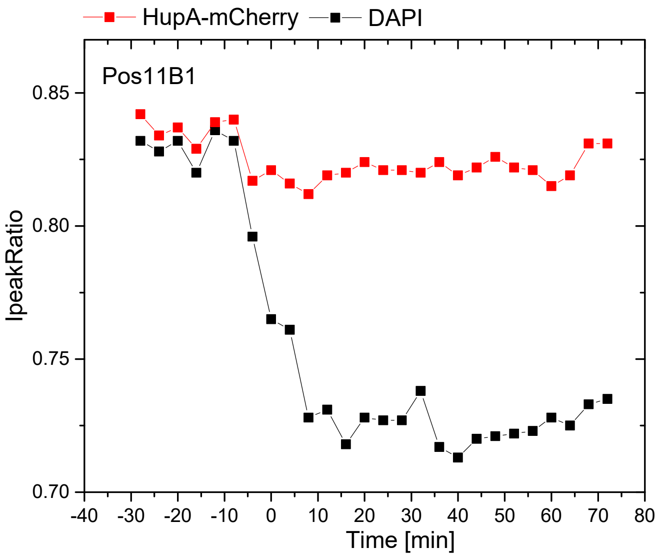

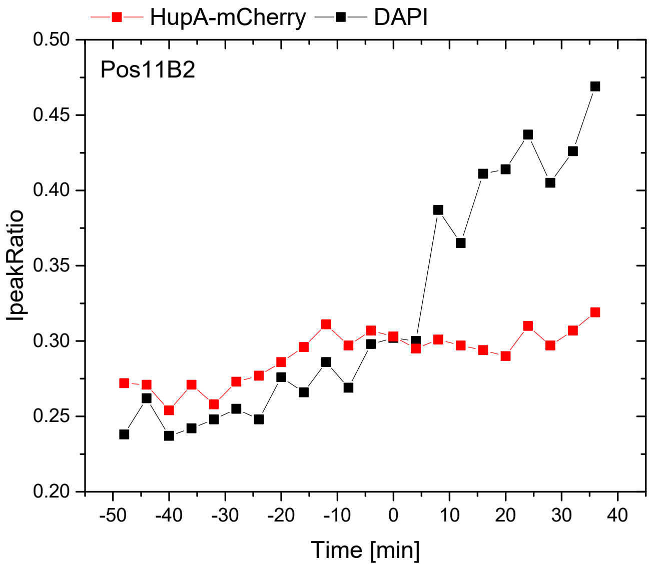

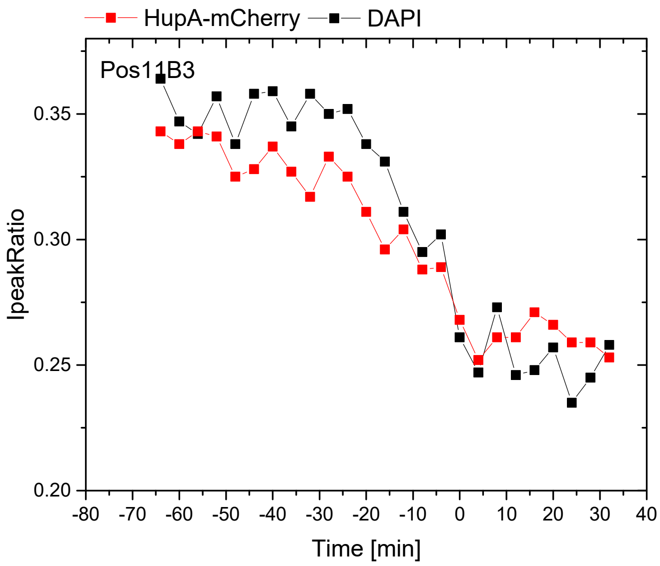

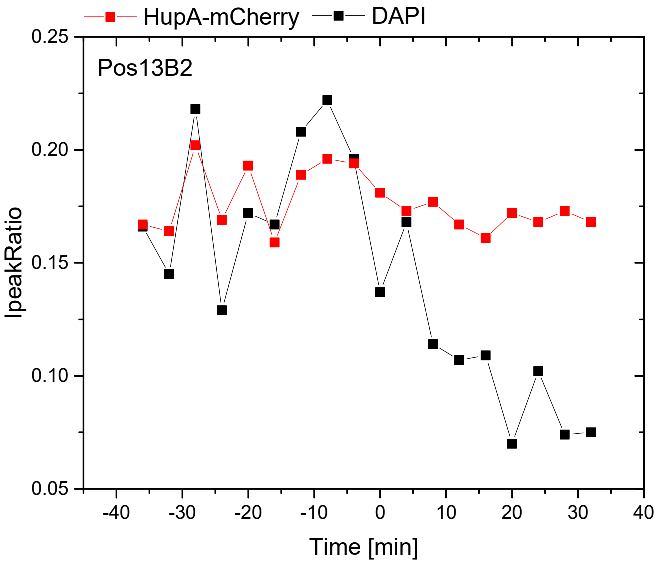

Strain MB16

HupA-mCherry and DAPI fluorescence; ZipA-GFP induced

IpeakRatio=(Normalized Intensity)/100

0 min. corresponds to time when ZipA-GFP label dissociates from the septum

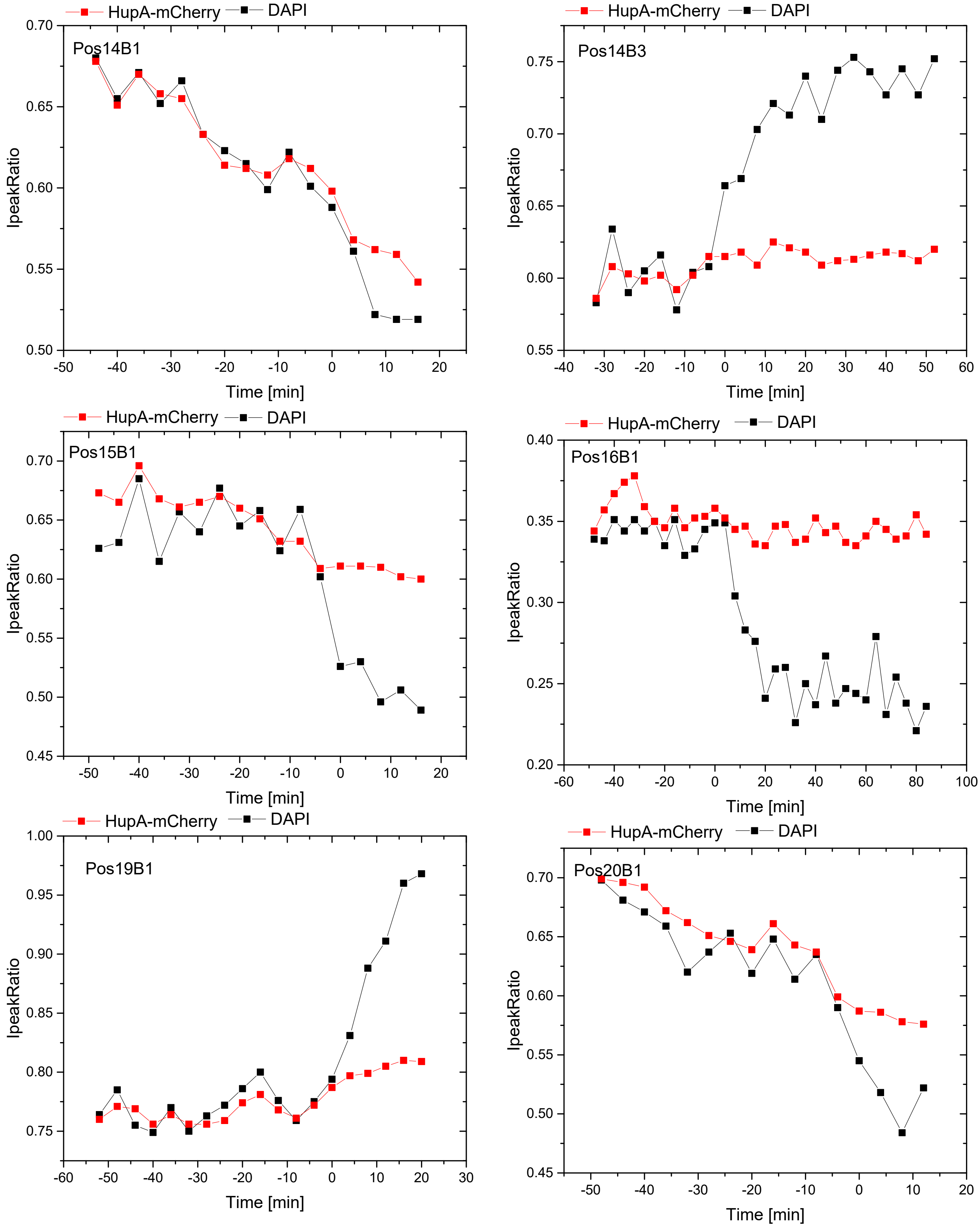

Strain MB16

HupA-mCherry and DAPI fluorescence; ZipA-GFP induced

IpeakRatio=(Normalized Intensity)/100

0 min. corresponds to time when ZipA-GFP label dissociates from the septum

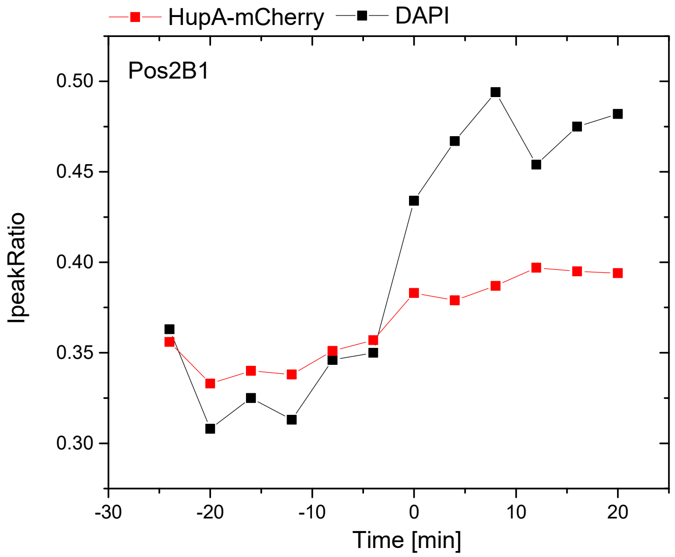

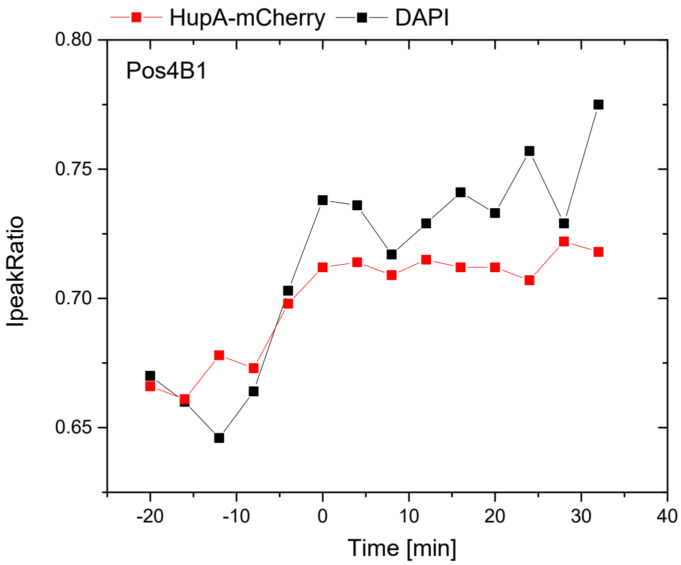

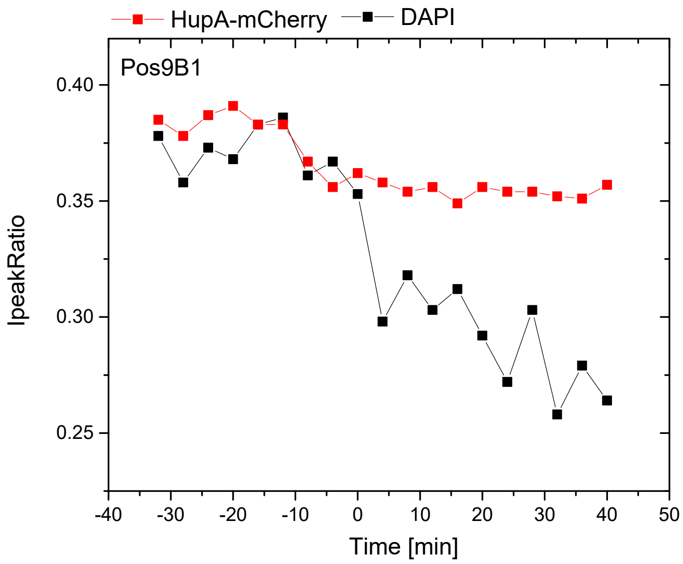

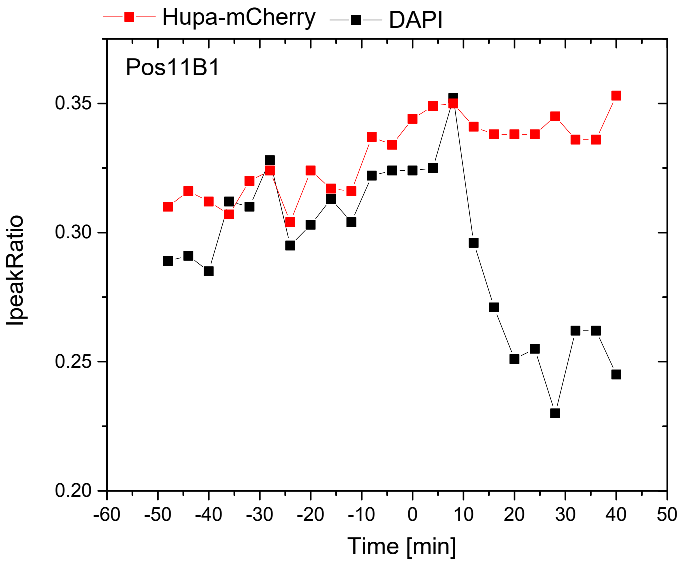

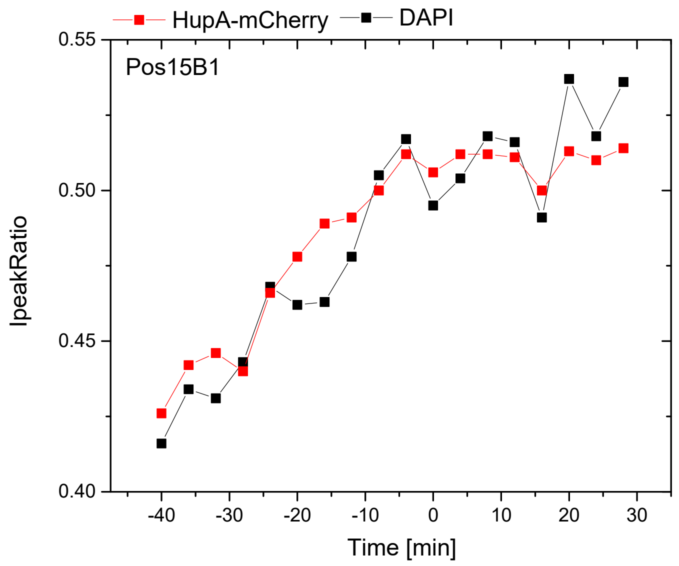

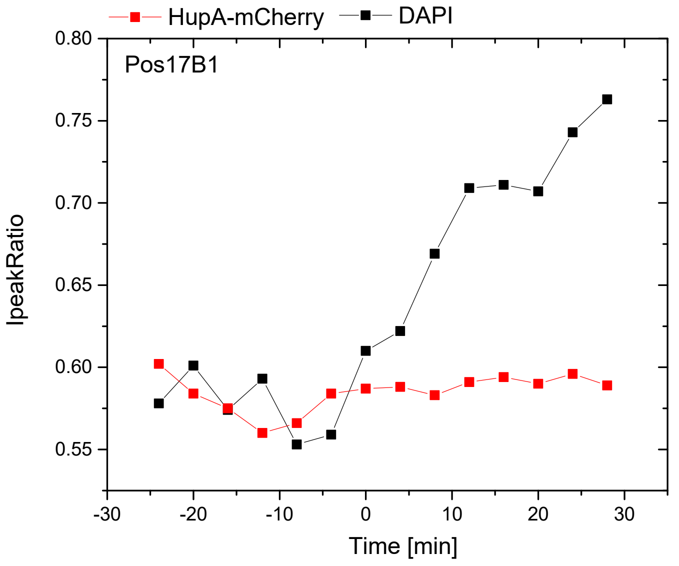

Supplement: S4 Dataset — (PDF) [file pgen.1006638.s016.pdf]
